# Epigenetic Modifier Supplementation Improves Mitochondrial Respiration and Growth Rates and Alters DNA Methylation of Bovine Embryonic Fibroblast Cells Cultured in Divergent Energy Supply

**DOI:** 10.3389/fgene.2022.812764

**Published:** 2022-02-24

**Authors:** Matthew S. Crouse, Joel S. Caton, Kate J. Claycombe-Larson, Wellison J. S. Diniz, Amanda K. Lindholm-Perry, Lawrence P. Reynolds, Carl R. Dahlen, Pawel P. Borowicz, Alison K. Ward

**Affiliations:** ^1^ USDA, ARS, U.S. Meat Animal Research Center, Clay Center, NE, United States; ^2^ Department of Animal Sciences, North Dakota State University, Fargo, ND, United States; ^3^ USDA, ARS, Grand Forks Human Nutrition Research Center, Grand Forks, ND, United States; ^4^ Department of Animal Sciences, Auburn University, Auburn, AL, United States

**Keywords:** cell growth, DNA methylation, embryonic fibroblasts, mitochondrial respiration, one-carbon metabolism

## Abstract

Epigenetic modifiers (EM; methionine, choline, folate, and vitamin B_12_) are important for early embryonic development due to their roles as methyl donors or cofactors in methylation reactions. Additionally, they are essential for the synthesis of nucleotides, polyamines, redox equivalents, and energy metabolites. Despite their importance, investigation into the supplementation of EM in ruminants has been limited to one or two epigenetic modifiers. Like all biochemical pathways, one-carbon metabolism needs to be stoichiometrically balanced. Thus, we investigated the effects of supplementing four EM encompassing the methionine–folate cycle on bovine embryonic fibroblast growth, mitochondrial function, and DNA methylation. We hypothesized that EM supplemented to embryonic fibroblasts cultured in divergent glucose media would increase mitochondrial respiration and cell growth rate and alter DNA methylation as reflected by changes in the gene expression of enzymes involved in methylation reactions, thereby improving the growth parameters beyond Control treated cells. Bovine embryonic fibroblast cells were cultured in Eagle’s minimum essential medium with 1 g/L glucose (Low) or 4.5 g/L glucose (High). The control medium contained no additional OCM, whereas the treated media contained supplemented EM at 2.5, 5, and 10 times (×2.5, ×5, and ×10, respectively) the control media, except for methionine (limited to ×2). Therefore, the experimental design was a 2 (levels of glucose) × 4 (levels of EM) factorial arrangement of treatments. Cells were passaged three times in their respective treatment media before analysis for growth rate, cell proliferation, mitochondrial respiration, transcript abundance of methionine–folate cycle enzymes, and DNA methylation by reduced-representation bisulfite sequencing. Total cell growth was greatest in High ×10 and mitochondrial maximal respiration, and reserve capacity was greatest (*p* < 0.01) for High ×2.5 and ×10 compared with all other treatments. In Low cells, the total growth rate, mitochondrial maximal respiration, and reserve capacity increased quadratically to 2.5 and ×5 and decreased to control levels at ×10. The biological processes identified due to differential methylation included the positive regulation of GTPase activity, molecular function, protein modification processes, phosphorylation, and metabolic processes. These data are interpreted to imply that EM increased the growth rate and mitochondrial function beyond Control treated cells in both Low and High cells, which may be due to changes in the methylation of genes involved with growth and energy metabolism.

## Introduction

Nutritional programming, often called early metabolic programming, establishes that early nutrition affects the development and function of organ systems, resulting in changes to programmed metabolism, immune function, neurodevelopment, and other physiological processes throughout life (Barker, 2007). In cattle, micronutrients such as B vitamins are synthesized by rumen microbes and are thought to be sufficient for embryonic and fetal development; however, recent data has shown an imbalance in the markers of B vitamin status, suggesting that this may not be the case (Crouse et al., 2019a). Furthermore, moderate changes in the rate of gain in beef heifers (pubertal virgin females that have reached approximately 60% of their mature body weight and average 0.45 kg per day of gain) during early gestation had reduced glucose and fructose concentrations in fetal fluids by day 50 of gestation in reduced gain heifers, suggesting that even moderate intake reduction in the dam manifests in nutrient restrictions to the embryo.

Throughout pregnancy, the whole and transitory energy requirements of macro- and micro-nutrients increase (Cai et al., 2021); however, during early gestation, the requirements of micronutrients, specific fatty acids, and amino acids and the interaction of these nutrients to meet the requirements for embryonic growth and differentiation are greater than the needs for whole-animal energy requirements (Koletzko et al., 2013; Marangoni et al., 2016; Cetin et al., 2019). Embryos undergo immense epigenetic remodeling during early development, and thus identifying specific micronutrient requirements *vs*. global energy requirements is needed. This epigenetic remodeling is driven by DNA methylation and epigenetic modifications of histone tails, including methylation, acetylation, and phosphorylation. These epigenetic mechanisms altogether regulate transcription, growth, and cell fate, leaving a “memory” on the embryo that will be carried forward through subsequent developmental stages (Sugden and Holness, 2002; Jaenisch and Bird, 2003; Waterland and Jirtle, 2004).

During early development, the embryo requires specific nutrients, such as epigenetic modifiers (EM; methionine, choline, folate, and vitamin B_12_), that play a key role in methylation reactions. One-carbon metabolism is the network of biochemical pathways that regulates amino acid metabolism, nucleotide synthesis, and epigenetic processes, such as methylation and demethylation of DNA (Ikeda et al., 2012; Clare et al., 2019). Methionine is the precursor for S-adenosylmethionine (SAM), which is the physiological methyl group donor for protein, RNA, and DNA methylation (Jeltsch, 2002). The DNA and histone methyltransferases use SAM as the primary methyl donor. The availability of SAM is directly influenced by diet as it is formed from methyl groups derived from choline, methionine, or methyl-tetrahydrofolate (Zeisel, 2011). Choline, *via* its metabolite betaine, and folate, *via* methyl-tetrahydrofolate, remethylate homocysteine to form methionine (Kim et al., 1994; Finkelstein, 2000). Vitamin B_12_ is a cofactor for methionine synthase, the enzyme responsible for folate-dependent methylation of homocysteine (Kalhan and Marczewski, 2012). The methionine–folate cycles are interconnected, and thus the metabolism of choline, folate, vitamin B_12_, and methionine are interrelated. Disturbances in one of these metabolic pathways due to deficiencies in EM are associated with compensatory changes in other parts of the methionine–folate cycle and in branching pathways, such as polyamine synthesis, nucleotide synthesis, the transulfuration pathway, phosphatidylcholine synthesis, and others (Zeisel, 2011; Clare et al., 2019). Deficiencies in the EM pool result in elevations in plasma homocysteine concentrations and are associated with impaired one-carbon metabolism resulting from protein malnutrition and deficiencies in folate and B_12_ (Castro et al., 2006). Research on the effects of EM supplementation or deficiency in ruminants on offspring health research has typically been limited to one or two epigenetic modifiers (Pagán et al., 2002; Sinclair et al., 2007; Martiniova et al., 2015), which makes our study of supplementing four EM to balance and drive the pathway a novel approach. In sheep fed purified diets limited in cobalt and sulfur (thereby limiting the rumen biosynthesis of vitamin B_12_ and methionine, respectively) from −60 to +6 days relative to insemination, the male offspring of methyl-donor-deficient ewes had an altered body composition by 22 months of age (Sinclair et al., 2007). In dairy cows, supplementation of individual methyl donors or combinations of methionine, choline, folate, and vitamin B_12_ has positively improved milk production, calf birth weight and height, amino acid flow to the calf, and nutrient profiles in milk (Preynat et al., 2009a, b; Batistel et al., 2017; Alharthi et al., 2018; Alharthi et al., 2019; Batistel et al., 2019; Palombo et al., 2021).

The objectives of this study aimed to identify how supplementation of four EM involved in the methionine–folate cycle impacted mitochondrial respiration, cell growth rate, and DNA methylation. Therefore, our data expands on current knowledge supplementing one or two EM by including the interactive roles of four EM at two different energy levels. These two energy levels were chosen to model data from heifers who were nutrient-restricted during early pregnancy and had reduced glucose in allantoic and amniotic fluid compared with the controls (Crouse et al., 2019). We hypothesized that EM supplemented to embryonic fibroblasts cultured in divergent glucose media would increase mitochondrial respiration and cell growth rate and alter DNA methylation, thus improving the growth parameters beyond Control treated cells.

## Materials and Methods

### Cells, Experimental Design, and Treatments

The bovine embryonic tracheal fibroblast cell line (EBTr; NBL-4; ATCC CCL-44) was purchased from the American Type Culture Collection (Manassas, VA, United States) and cultured in Eagle’s minimum essential medium (EMEM: Sigma, St. Louis, MO, United States) supplemented with 10% fetal bovine serum (Thermo Fisher Scientific, Waltham, MA, United States), 1% penicillin–streptomycin (Thermo Fisher Scientific), and 0.11 g/L Na pyruvate (Sigma). D-Glucose (sigma) was added to achieve final glucose concentrations of 1 g/L (Low) or 4.5 g/L (High). The control medium contained basal concentrations of EM as found in EMEM media: folate (0.001 g/L), choline (0.001 g/L), vitamin B_12_ (4 μg/L), and methionine (0.015 g/L). Epigenetic modifiers (folic acid, choline chloride, vitamin B_12_, and L-methionine) were added to the media at 2.5, 5, or 10 times (×2.5, ×5, or ×10, respectively) the concentrations in the control medium, except for methionine, which was included at ×2 for all supplemented treatments to prevent methionine toxicity. These EM concentrations were modified after Cho et al. (2012), in which folate, choline, and vitamin B_12_ were supplemented at ×5 control and methionine was limited to ×1.8 control. To complete the four treatments, we employed one-half and 2 times ×5 (×2.5 and ×10, respectively) for doubling effects and analysis *via* polynomial contrasts with increasing EM concentrations. Therefore, the experiment was a completely randomized design with two glucose levels [Low and High] × 4 EM levels [control, ×2.5, ×5, and ×10] under a factorial arrangement of treatments. For all analyses, the cells were cultured in 10-cm cell culture dishes in a humidified incubator at 37°C and 5% CO_2_ and passaged three times in treatment media once the cells reached 80% confluence in the dish (average of 2 days) before being plated for further analyses.

### Cell Growth and Proliferation Analyses

Cells were plated in triplicate at a cell density of 1.8 × 10^3^ cells per well onto one of six Seahorse XFe-24 microplates (Agilent, Santa Clara, CA, United States). The cells were placed in a humidified incubator (37°C, 5% CO_2_) for either 1, 12, 24, 36, 48, or 72 h. At the designated time, the plates were removed from the incubator, after which time the media were aspirated and the cells were fixed in 10% neutral-buffered formalin (Sigma). Antigen retrieval was performed in 10 mM sodium citrate buffer, pH 6, with 0.05% Tween 20 in a 2100 Retriever (Electron Microscopy Sciences, Hatfield, PA, USA). The wells were treated for 1 h with 10% normal goat serum (Vector Laboratories, Burlingame, CA, USA) to block nonspecific binding. For proliferation analysis, each well was stained with rabbit anti-Ki67 (Abcam, Cambridge, UK) for 1 h and fluorescently labeled with CF633 goat anti-rabbit secondary antibody (Biotium, Fremont, CA, United States) for 1 h. To counterstain all nuclei, cells were treated with 4,6-diamidino-2-phenylindole (DAPI; Life Technologies, Grand Island, NY, United States). MosaiX images were captured with a Zeiss Imager M2 epifluorescence microscope using a ×5 objective and AxioCam HRm camera and were analyzed using ImagePro Premiere software (Media Cybernetics, Silver Spring, MD, United States) for the total cell number (DAPI-stained cells) and proliferating cell number (Ki67-stained cells). To calculate cell growth as a percent of originally plated cells, the average of the hour 1 value (cell numbers at hour 1 = 1,615 ± 23 cells) or the number of cells attached to the wells for each treatment was averaged to equal 100%. The number of cells at each timepoint within a treatment from hour 12 to 72 was then compared against hour 1 to determine cell growth as a percent of originally plated cells. Cell proliferation was calculated as the labeling index (% of cells stained by Ki67).

### Mitochondrial Respiration

Oxygen consumption rate was measured using a Seahorse XFe24 analyzer (Agilent, Santa Clara, CA, United States). EBTr cells were passaged three times in treatment media before being plated onto Seahorse XFe24 microplates at 5 × 10^4^ cells per well in 150 µl of media to allow cells to attach to the bottom of the well. At 1 h after plating, an additional 150 µl of treatment media was added carefully to the well so as not to disturb the attached cells. The cells were cultured in Seahorse XFe24 microplates for 18–24 h. Immediately prior to analysis, the treated media was exchanged for XF Assay Dulbecco modified Eagle’s medium (pH = 7.4) supplemented to contain 4.5 mM glucose (Agilent), 1 mM pyruvate (Agilent), and 2 mM glutamine (Agilent), and the plates were incubated at 37°C in a CO_2_-free incubator for 1 h. Baseline oxygen consumption rates were measured three times before sequentially adding Oligomycin 12.64 µM (Agilent), FCCP 10 µM (Agilent), and Rotenone/Antimycin A 10 µM (Agilent). The read parameters were as follows: 1 min of mixing and 2 min of waiting after mixing, followed by 2 min to read, completed three times per measurement. Immediately following analysis, the XF assay medium was removed from the wells, and the cells were fixed in 10% neutral-buffered formalin (Sigma). Calculation of total cell number for normalizing oxygen consumption for mitochondrial analysis was conducted using the same methodology as previously described for total growth rate. The mitochondrial respiration parameters were measured as O_2_ consumption per picomole per minute per cell scaled to 10,000 cells. The parameters measured included the following: basal respiration, O_2_-linked ATP synthesis (basal respiration - O_2_ consumption after injection with oligomycin), maximal respiration, reserve capacity (maximal respiration–basal respiration), proton leak (O_2_ consumption after injection with oligomycin–O_2_ consumption after injection with rotenone/antimycin A), and non-mitochondrial respiration. Each reported mean for the measurement is the average of three reads per parameter across each treatment. The intra-plate coefficient of variation (CV) was 6.39%, and the across-plate CV was 12.53%.

### Transcript Abundance of Methionine–Folate Cycle Enzymes

Cells were plated into T-75 flasks (*n* = 3/treatment) and grown to 90% confluency. Once target confluence was reached, cells were collected, and RNA was extracted and purified using the Aurum Total RNA Mini Kit (Bio-Rad, Hercules, CA, USA). Total RNA was quantified using the Take3 module of a Synergy H1 Microplate Reader (BioTek, Winooski, VT, USA). Moreover, 1,000 ng/μl RNA was used to synthesize complementary DNA for each sample by utilizing iScript cDNA Synthesis Kit (Bio-Rad, Hercules, CA, United States). Taqman™ hydrolysis probes (Applied Biosystems, Grand Island, NY, United States) were purchased from ThermoFisher (Waltham, MA; Supplementary Table S1). Optimal cDNA dilutions were determined by primer validation for each gene: methionine adenosyltransferase 1A, 2A, and 2B (MAT1A, MAT2A, and MAT2B); DNA methyltransferase 1, 3A, and 3B (DNMT1, DNMT3A, and DNMT3B), S-adenosylhomocysteine hydrolase, methionine synthase (MTR), and betaine-homocysteine S-methyltransferase. For each sample, gene expression was quantified in triplicate using a 7500 Fast Real-Time PCR System (Applied Biosystems), with Taqman™ Fast Advanced Master Mix (Applied Biosystems), with 20-µl total reaction volume for all genes. Relative mRNA expression was calculated using the 2^−ΔΔCt^ method (Livak and Schmittgen, 2001) with succinate dehydrogenase, as it was the most stable of all reference genes analyzed (Supplementary Table S1). The intra-plate CV was 0.12%, and the across-plate CV was 0.98%.

### Reduced Representation Bisulfite Sequencing

Based on previous analysis with mitochondrial respiration and cell proliferation, reduced representation bisulfite sequencing (RRBS) was only conducted on Low and High: control, ×2.5, and ×5 treatments. Cells were plated into T-75 culture flasks grown to 90% confluency. Once the target confluence was reached, cells were collected and cell pellets (1 × 10^6^) were frozen in cryo vials (*n* = 3 tubes/trt) and shipped to the University of Minnesota Genomics Center. The DNA was extracted from the cell pellets with the DNeasy Blood and Tissue Kit (Qiagen, Hilden, Germany) and quantified using the PicoGreen DNA Quantification Kit (Invitrogen). Libraries (*n* = 18) were prepared from extracted DNA (20 ng/μl) using the NuGEN Ovation RRBS Methyl-Seq Kit (Tecan Genomics, Redwood City, CA, United States). The libraries were bisulfite-converted and PCR-amplified prior to sequencing with NovaSeq S Prime in the 150 paired-end reads mode (Illumina, San Diego, CA, USA).

### Statistical Analysis

Cell growth was analyzed after normalization to the number of cells plated per treatment at hour 1 set to 100% using PROC MIXED (SAS, CARY, NJ, United States) with glucose, EM, and their interaction as fixed effects. Cell growth was analyzed at the following timepoints: 12, 24, 36, 48, and 72 h post-plating. Labeling index (cell proliferation) was analyzed at six timepoints: 1, 12, 24, 36, 48, and 72 h post-plating.

Gene expression data are presented as a fold change relative to High Control which has been set to 1.00. Gene expression and mitochondrial respiration parameters were analyzed with PROC GLM of SAS 9.4 for glucose, EM, and their interaction.

Polynomial contrasts were performed within the Low and High glucose levels to determine whether increasing EM affected the cell growth rate, proliferation, mitochondrial respiration, or gene expression. The analysis included linear, quadratic, and cubic polynomial contrasts to determine if the response variables measured increased or decreased with increasing EM concentrations within a glucose level. To account for unequal spacing of EM treatments, contrast coefficients were determined using PROC IML and analyzed with the GLM procedure for effects of EM supplementation within the glucose level. All *P*-levels less than or equal to 0.05 were considered significant. Visualization and description of contrasts are included for those with significant contrasts in the polynomial contrast graphs (Supplementary Material).

### RRBS Data Quality Control and Differential Cytosine Methylation Analyses

An average of 14.5 M reads was generated for each sample (three samples per treatment). The quality control of raw reads was assessed using FastQC v0.11.8 (Andrews, 2010) and MultiQC v1.9 (Ewels et al., 2016) software. Adapter trimming and low-quality bases (Phred score <20) were filtered out using Cutadapt v.2.10 (Martin, 2011). NuGEN’s diversity trimming was carried out to remove diversity adaptors as suggested by NuGEN’s analysis guide (https://github.com/nugentechnologies/NuMetRRBS). The trimmed reads were mapped to the bovine reference genome (UCSC—bosTau8, Illumina IGenome). Nextflow methylseq pipeline v1.5 (Ewels et al., 2020) was used to perform read mapping and methylation call through Bismark v0.22.3 software. Only cytosines in a CpG context were retrieved and analyzed. Reads containing CpGs with more than 99.9th percentile coverage and less than 10 counts in every sample were filtered out to avoid biases due to varying sequencing depth.

Ten contrasts were tested as follows: (1) High ×5 *vs*. High CON, (2) High ×2.5 *vs*. High CON, (3) High ×5 *vs*. High ×2.5, (4) Low ×5 *vs*. Low CON, (5) Low ×2.5 *vs*. Low CON, (6) Low ×5 *vs*. Low CON, (7) High ×5 *vs*. Low ×5, (8) High ×2.5 *vs*. Low ×2.5, (9) High CON *vs*. Low CON, and (10) High ×5 *vs*. Low CON. Differentially methylated cytosines (DMC) were identified using edgeR (Robinson et al., 2010; Chen et al., 2017) and considered significant for each of the contrasts when the *p*-value cutoff <0.01. Differentially methylated or demethylated cytosines were defined based on the sign of the log2 fold change. The gene transcriptional start site closest (no specific cutoff) to each CpG was used to annotate all DMCs. To identify the biological processes underlying the genes harboring DMCs, we performed a functional analysis separately for each gene list of the 10 contrasts. The gene over-representation analysis was performed on ShinyGO v0.61 (Ge et al., 2020) using the *Bos taurus* annotation as background. Significant BPs within each gene list were retrieved after multiple testing adjustments when false discovery rate (FDR) <0.05. To identify over-represented BPs within and between contrasts, we overlapped the BPs retrieved as significant in each contrast list. Overlapping BPs were identified and visualized based on the -log (FDR) using the R-package pheatmap v1.0.10 (Kolde, 2019).

## Results

### Cell Growth

**FIGURE 1:**
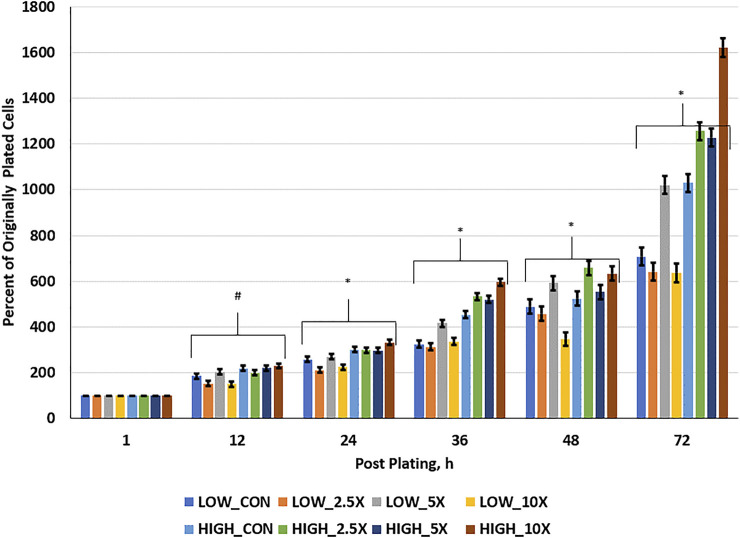
Cell growth rate is increased in High cells with increasing epigenetic modifiers (EM) and to ×5 in Low cells. Three replicates per treatment per timepoint were measured. The error bars represent the average standard error of the mean within the glucose level. # indicates a tendency (0.05 < *p* < 0.10) for an interaction of glucose × EM. * indicates a significant (*p* ≤ 0.05) interaction of glucose × EM.

Cell growth rate data are presented in Figure 1 and Supplementary Table S2. Cell growth at hour 12 was greater in High compared with Low cells. At 24 h post-plating, all High treated cells had a greater percent of originally plated cells compared with Low Control, ×2.5, and ×10; however, Low ×5 was greater than Low ×2.5 and ×10 and equal to High ×2.5 and ×5. At 36 h post-plating, patterns of divergence of growth rate of cells within treatments occur and remain similar through h 72. Interactions of glucose level and EM occur where High ×10 cells are growing at greater rates than all other treated cells, with High ×2.5 and ×5 being equal to one another and greater than High Control (*p* < 0 0.01). Furthermore, in the Low cells, Low ×5 treated cells had a greater percentage of originally plated cells than all other Low treated cells and, by 72 h post-plating, had an equal number of originally plated cells compared with High Control, suggesting a compensatory growth beyond the Low Control to equal that of the High Control. Finally, at 72 h post-plating, Low ×2.5 and ×10 showed no improvement in growth compared to the Low Control (*p* ≥ 0.26); however, High 10× showed the greatest cell growth at 1,620% of the originally plated cells (*p* < 0.01).

### Cell Proliferation

**FIGURE 2:**
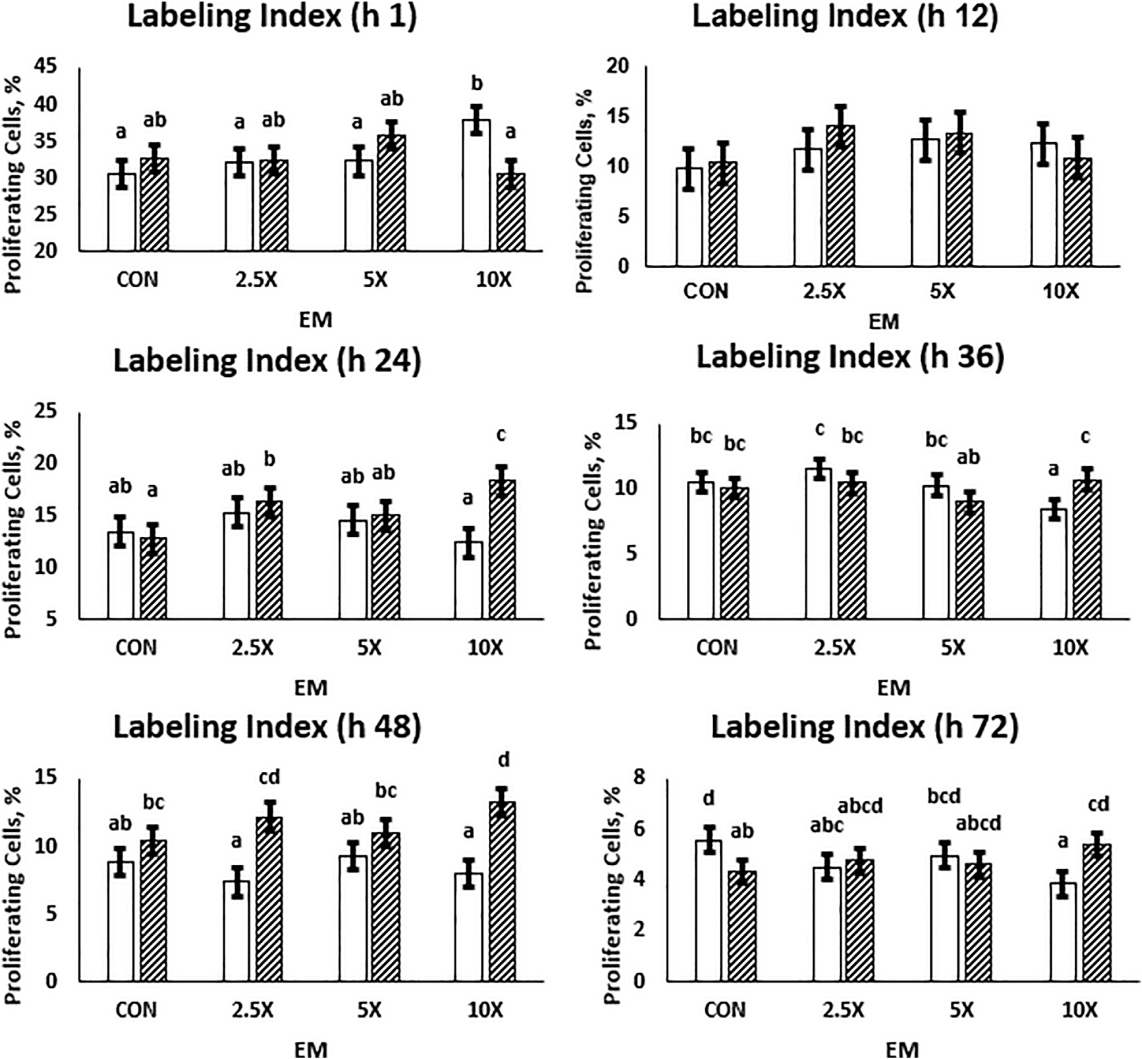
The cell proliferation responses are dependent on the time of measurement and decrease as cells reach confluency. High glucose cells are generally greater than Low cells, and the labeling index in High cells across each treatment is representative of growth rates. Low glucose cells are represented by the solid white bar. High glucose cells are represented by the dashed bar. Means without a common superscript differ in glucose × epigenetic modifiers (*p* ≤ 0.05). Three replicates per treatment per timepoint were measured. Error bars represent the average standard error of the mean within a glucose level.

Cell proliferation data are presented in Figure 2 and Supplementary Table S3. Overall, the labeling index is a single-point measurement in time and decreased from an average of 33% proliferating cells to 4.75% from hour 1 to 72 as cells became confluent in the wells. At h 24, 36, 48, and 72, the cell proliferation data followed a similar pattern. In High treated cells, the proliferation increased (*p* ≤ 0.05) with increasing supplementation level. At h 24, High ×10 treated cells had 43% more proliferating cells than High Control. In Low cells, the percent of proliferating cells remained similar or decreased (*p* ≤ 0.10) as the EM supplementation level increased. By h 72, Low ×10 treated cells had 31% fewer proliferating cells compared with Low Control at the same time point (*p* = 0.01). At all timepoints, Low ×10 proliferation was decreased compared with all other treatments. While all High treated cells and Low ×5 reached at least 90% confluence in the well at h 72 and thus proliferation decreased due to confluence, Low Control, ×2.5, and ×10 had not reached those levels of confluence. Low Control, ×2.5, and ×10, which averaged a 660% increase in cells (Figure 1), had a decreased percent of proliferating cells at h 48 and 72 compared with the other treatments.

### Mitochondrial Respiration

**FIGURE 3:**
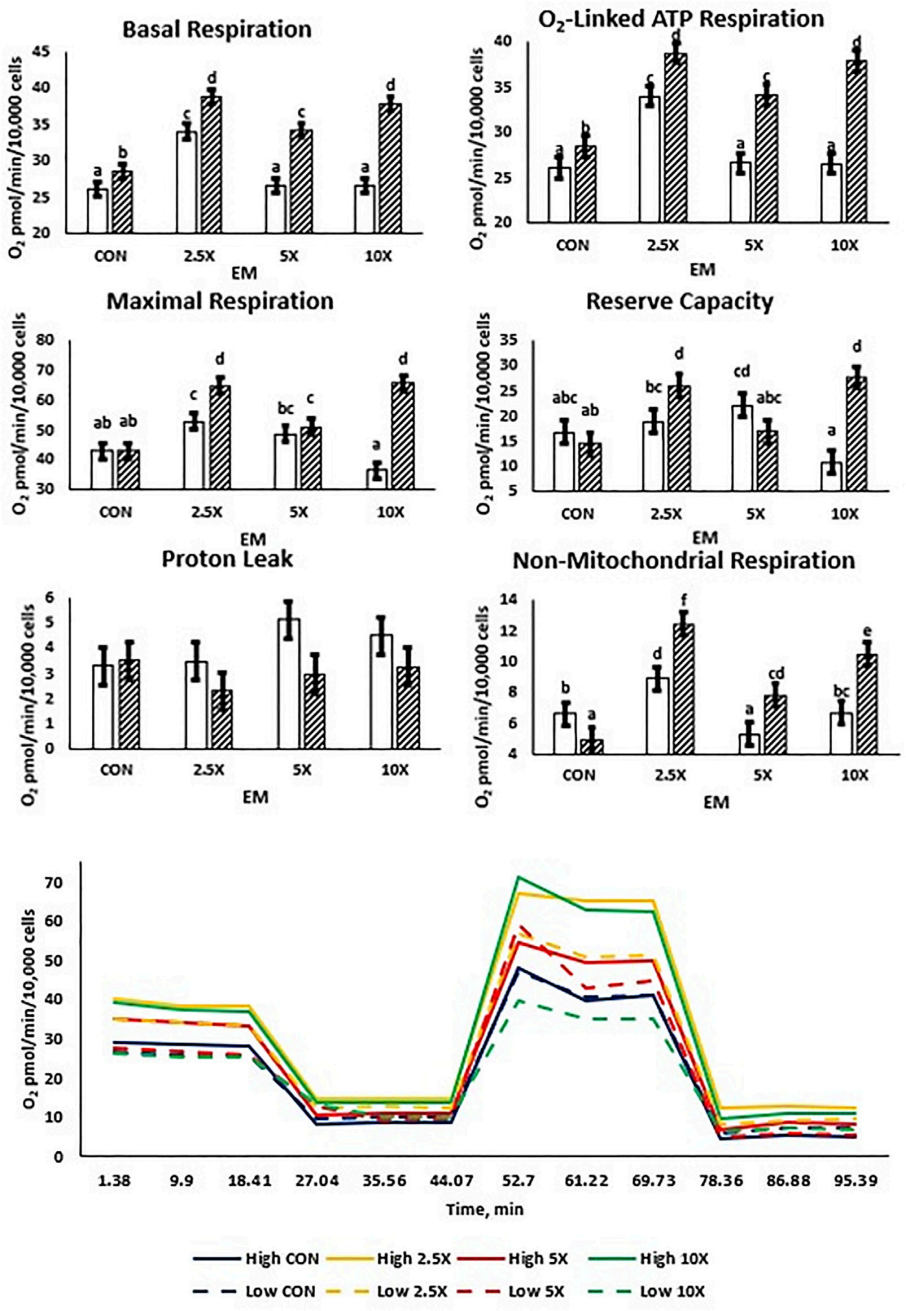
Mitochondrial respiration increases cubically with supplementation of epigenetic modifiers (EM) in High but not in Low cells. Basal respiration and O_2_-linked respiration increase to ×2.5 in Low, but reserve capacity increases to ×5 in Low. Low glucose cells are represented by the solid white bar. High glucose cells are represented by the dashed bar. Means without a common superscript differ in glucose × EM (*p* ≤ 0.05). Three replicates per treatment were measured (three technical replicates per biological replicate). Error bars represent the average standard error of the mean within a glucose level at each measurement.

All mitochondrial respiration data are presented in Figure 3 and Supplementary Table S4. Both High and Low treated cells exhibited cubic and quadratic responses. Basal respiration, O_2_-linked ATP synthesis, maximal respiration, and reserve capacity followed similar cubic patterns in High cells increasing (*p* ≤ 0.04) from Control to ×2.5, decreasing at ×5, and subsequently increasing the oxygen consumption at ×10 to be equivalent to that of ×2.5. Low glucose cells were affected quadratically or cubically, increasing (*p* ≤ 0.04) to ×2.5 and decreasing in oxygen consumption to the ×5 and ×10 EM supplementation level. There were no differences in proton leak between glucose and EM supplementation (*p* ≥ 0.06) in either the Low or High treated cells.

### Transcript Abundance of Methionine–Folate Cycle Enzymes

**FIGURE 4:**
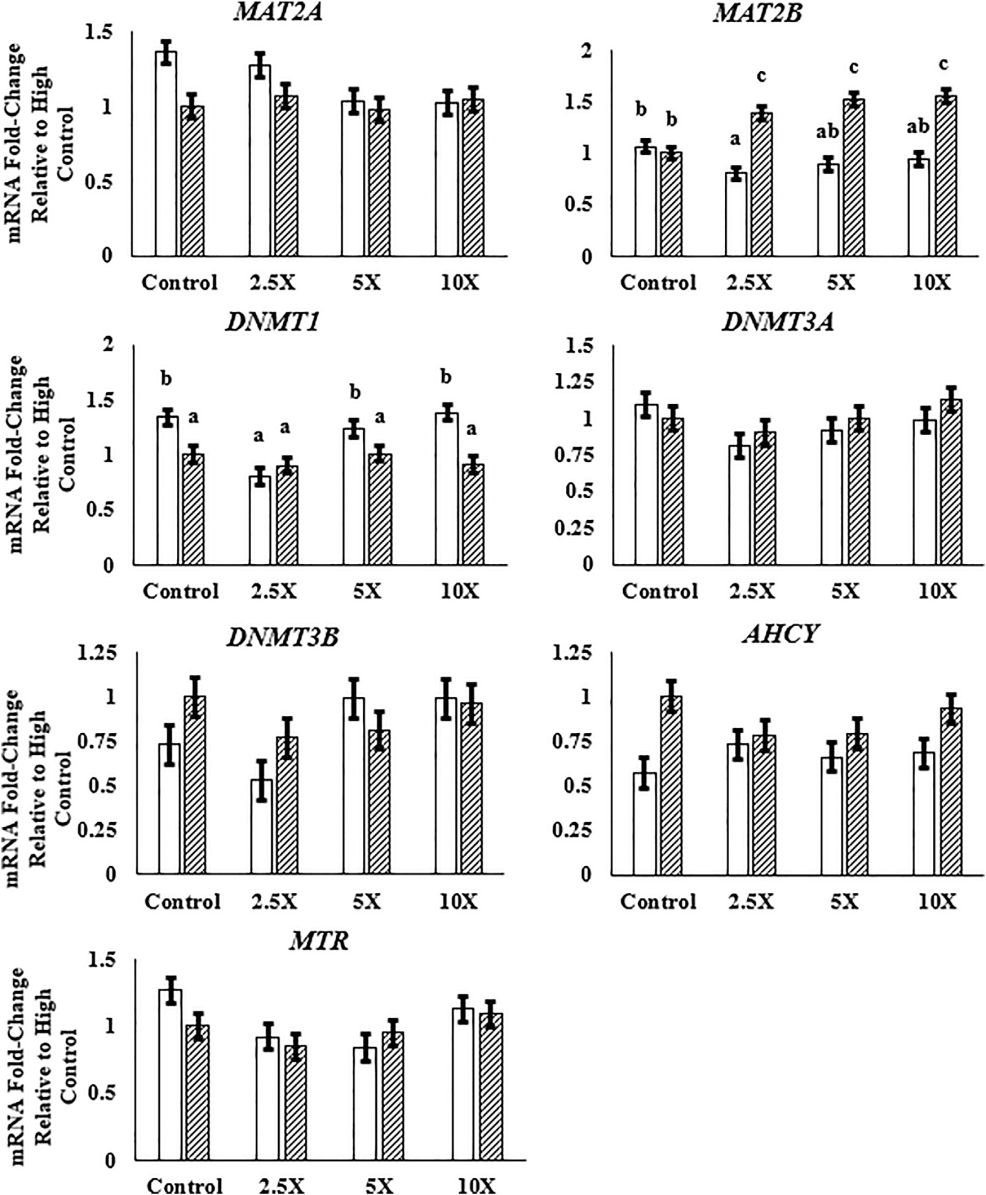
The expression of *MAT2B* is greater with epigenetic modifier (EM) supplementation in High cells, and *DNMT1* expression is lower in High compared with Low cells. High glucose cells are represented by the dashed bar. MAT2A, methionine adenosyltransferase 2A; MAT2B, methionine adenosyltransferase 2B; DNMT1, DNA methyltransferase 1; DNMT3A, DNA methyltransferase 3A; DNMT3B, DNA methyltransferase 3B; AHCY, S-adenosylhomocysteine hydrolase; MTR, methionine synthase. Low glucose cells are represented by the solid white bar. High glucose cells are represented by the dashed bar. Means without a common superscript differ in glucose × EM (*p* ≤ 0.05). Only means that are significant for the glucose × EM interaction will have mean separation. Three replicates per treatment per timepoint were measured. Error bars represent the average standard error of the mean within a glucose level.

Data in this section are presented in Figure 4 and Supplementary Table S5. The expression of *MAT2A* and *MAT2B*, genes encoding enzymes responsible for the synthesis of SAM, was differentially respondent to the EM treatment. The relative expression of *MAT2A* decreased linearly (*p* = 0.02) in Low cells, whereas the relative expression of *MAT2B* increased quadratically (*p* = 0.02) in High cells from High Control to High ×10. Among the genes coding methyltransferase enzymes, DNMT1, DNMT3A, and DNMT3B, only the *DNMT1* relative mRNA expression was altered, with a decreased expression in High compared with Low cells. Both *de novo* methyltransferases were unaffected (*p* ≥ 0.07) by glucose or EM supplementation. Methionine synthase was altered by EM supplementation, such that the mRNA expression decreased (*p* = 0.03) to the ×2.5 and ×5 EM supplementation level compared with the Control treated cells.

### Differentially Methylated Cytosines

**FIGURE 5:**
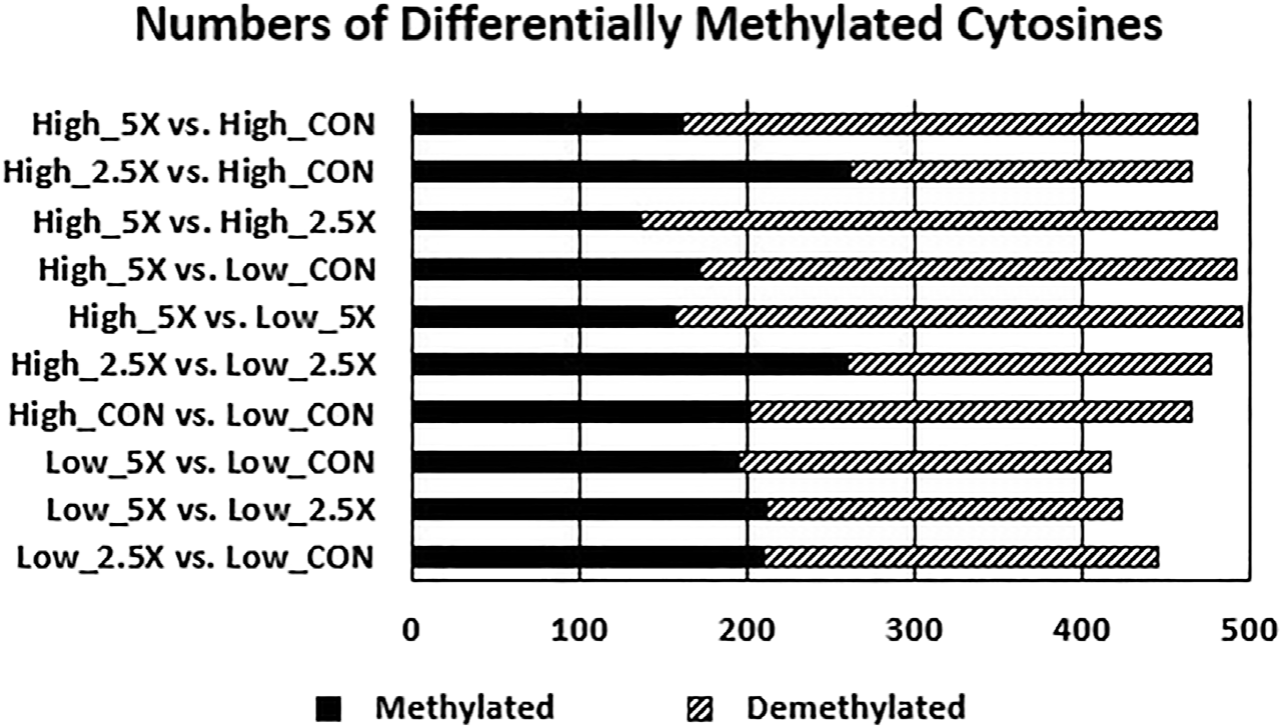
Increasing epigenetic modifier (EM) supplementation decreases the number of methylated cytosines (*p* < 0.01) of embryonic tracheal fibroblast cells across treatments. Data are presented as contrasts between treatments where the number of methylated and demethylated cytosines of the first treatment listed are compared against the second (*i*.*e*., the number of differentially methylated and demethylated cytosines in the High ×5 treatment compared with the High Control treatment showing a greater number of demethylated cytosines compared with methylated cytosines in High ×5 compared with High Control). The differentially methylated and demethylated cytosines were defined based on the sign of the log2 fold change. The number of differentially methylated cytosines are represented by the solid black bar. The number of differentially demethylated cytosines are represented by the dashed bar.

The data presented in this section are contrasts of DMC (*p* < 0.01) between treatments and are presented in Figure 5 and the differentially methylated cytosines in the Supplementary Material. Contrasts comparing High ×2.5 *vs*. High Control and High ×2.5 *vs*. Low ×2.5 had a greater number of methylated cytosines than demethylated cytosines, suggesting genome hypermethylation. The contrast comparing Low ×5 *vs*. Low Control had equal numbers (*n* = 212) of differentially methylated and demethylated cytosines. The remaining contrasts showed more cytosines that were differentially demethylated.

### Overlapping Biological Processes

**FIGURE 6:**
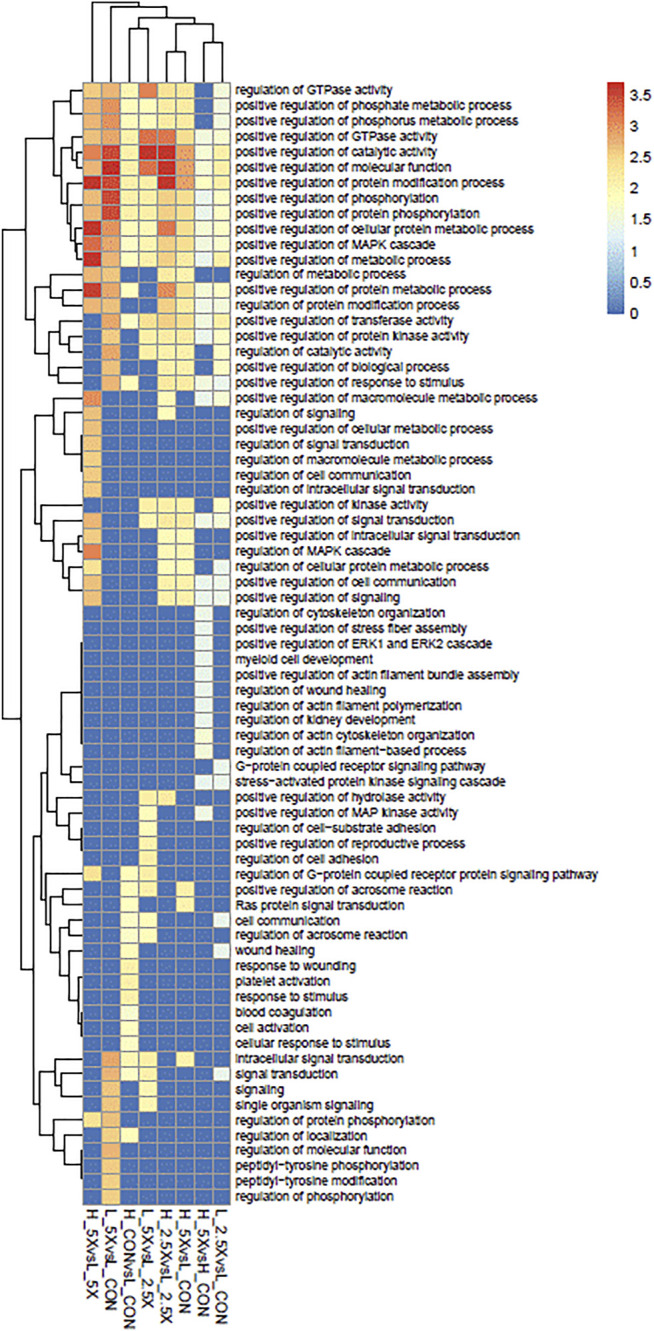
Over-represented biological processes (BPs) of genes with differentially methylated cytosines from embryonic tracheal fibroblast cells treated with different levels of glucose and epigenetic modifiers. The plot is color-coded based on -log10 (false discovery rate) of different BPs.

The functional analyses based on the gene lists retrieved 73 significantly over-represented BPs across all comparisons. The genes harboring DMCs were underlying mainly regulatory functions. Among the 73 gene ontology terms, 49 had the word “regulation”. Among the BPs, we had a positive regulation of different MAPK cascades, phosphorylation, and metabolic process. These BPs were shared across all treatments, except the High ×2.5 *vs*. High Control and High ×5 *vs*. High ×2.5 comparisons, which did not show any significant BP. Figure 6 shows the significantly over-represented BPs within and across all comparisons. The biological processes from all the contrasts are in the biological processes all contrasts of the Supplementary Material.

## Discussion

The objectives of this study were to identify how supplementation of four EM involved in the methionine–folate cycle impacted mitochondrial respiration, cell growth rate, and DNA methylation. Currently, the mechanisms of effects of EM in beef cattle are limited, and thus this study utilized techniques to measure the growth rate, cell proliferation, mitochondrial respiration, and DNA methylation to shed light on the potential mechanisms driving fetal programming in response to EM supplementation. This study is unique in two ways: (1) inclusion of four metabolites and cofactors involved in the methionine–folate cycle pathway and (2) using a bovine model to investigate the effects of supplementing multiple EM on embryonic cell growth, mitochondrial respiration, and cytosine methylation.

In this study, measurements across glucose levels saw expected increases in growth rate and mitochondrial respiration due to the greater energy provided by glucose; however, there were fewer differences in labeling index across glucose levels, and the total percent of proliferating cells decreased as cells became confluent in the well. High treated cells and Low ×5 reached at least 90% confluency; however, Low Control, ×2.5, and ×5 never reached a high percentage of confluency yet still had a decreased labeling index at hour 48, and Low ×10 had the least percentage of proliferating cells at h 72. While the decrease in labeling index for the treatments with the greatest growth rates is most affected due to cell senescence, the decrease in proliferation of Low cells may be due to glucose being consumed and being insufficient for growth for the 72-h period, thus resulting in decreased cell proliferation and growth rates. Additionally, Low ×5 may respond similarly to the effects seen with growth rates in High treated cells where nutrients may be partitioned more efficiently for growth, and thus solidifying that use of these metabolites for positive responses is energy dependent. More research would have to be conducted using labeled metabolites to determine pathway directionality and confirm these hypotheses.

Although labeled as Low and High glucose, the difference of 3.5 g/L between the two treatments is a moderate increase in glucose level, which was designed to mimic small (1.63 *vs*. 1.31 mM and 1.79 *vs*. 1.34 mM: *p* ≤ 0.05) changes in glucose concentrations seen in the allantoic and amniotic fluids of control *vs*. nutrient-restricted heifers (Crouse et al., 2019a). In this study, the measurements of growth rate and mitochondrial respiration improved when given 2.5, 5, and 10 times the EM supplementation of the Control treated cells in High glucose medias; however, in the Low glucose media, growth rate and mitochondrial respiration improved in the ×5 treatment but declined drastically at the ×10 level. Proliferating cells preferentially utilize glucose for energy *vs*. the electron transport chain, which will increase the cellular reactive oxygen species (Antico Arciuch et al., 2012). The data reported within demonstrated cubic responses in both O_2_-linked ATP respiration and non-mitochondrial respiration, suggesting that responses in growth rate may be due to both glycolytic and respiratory ATP production. The one-carbon metabolism pathway also ties to cellular redox quenching through the synthesis of cysteine (Clare et al., 2019), which is a precursor of glutathione, the electron donor for the glutathione peroxidase reaction to reduce radicals such as hydrogen peroxide to water. In mice embryonic fibroblast (3T3-L1) cells, oxidative phosphorylation increases twofold in proliferating cells compared to quiescent cells (Yao et al., 2019). In High glucose cells, supplementation with ×2.5 and ×10 EM increased the oxygen consumption by at least 1.5-fold; however, in the Low glucose cells, increasing the EM only increases oxygen consumption at the ×2.5 supplementation level. In both studies, EBTr and 3T3-L1 are embryonic fibroblasts, and both show similar increases in oxidative phosphorylation and oxygen consumption in cells that are proliferating and growing compared to those of a slower growth rate and lower proliferating percentage. Acosta et al. (2016) reported that embryos collected from dairy cows supplemented with methionine had decreased 5-methylcytosine methylation compared with embryos flushed from cows fed a control diet. Furthermore, these embryos had greater lipid accumulation, which is an energy source for the developing embryo (Sturmey et al., 2009).

To evaluate how changes in DNA methylation may be due to differences in the function of the methionine–folate cycle, the expression of enzyme-coding genes responsible for the generation of SAM, DNA methyltransferases, or remethylation of homocysteine to methionine was measured. Methionine adenosyltransferase 1A and betaine-homocysteine methyltransferase transcripts were not expressed. This was to be expected as these enzymes are limited to function in hepatic tissues (Nordgren et al., 2011; Ji et al., 2012) and the cells utilized in this experiment were tracheal fibroblasts. Methionine adenosyltransferase 2A is responsible for synthesizing SAM from ATP and methionine, while *MAT2B* encodes a regulatory protein for MAT2A (Nordgren et al., 2011). Epigenetic modifier supplementation did not affect the expression of *MAT2A*; however, *MAT2B* expression increased in High treated cells with increasing EM supplementation level. Although regulatory, there were no changes in the expression of *MAT2A*, which would suggest that the change in *MAT2B* expression did not alter the function of MAT2A.

When evaluating methyltransferases, *DNMT3A* and *DNMT3B* expression was not altered by glucose or EM supplementation. Both DNMT3A and DNMT3B are *de novo* methyltransferases responsible for the methylation of early embryos and play critical roles in proper development (Okano et al., 1999). The cells utilized in this experiment are tracheal fibroblasts which would be collected from later-stage embryos. Immediately following fertilization, there is a wave of global demethylation of paternal DNA followed by a slower but complete demethylation of maternal DNA to the 8-cell stage (Morgan et al., 2005; Dobbs et al., 2013; Jiang et al., 2018). The global epigenetic remodeling events take place earlier in embryonic development, and thus a lack in the change in expression of *DNMT3A* and *DNMT3B* is supported by the later stage of embryonic development that this immortalized cell line originated from. In beef cattle, Crouse et al. (2019b) reported that, even at day 50 of gestation, there are changes in the transcript abundance of genes involved in the epigenetic reprogramming of fetal tissue accretion, function, and metabolism. In cattle, organogenesis occurs until approximately day 45 to 50 of gestation (Winter et al., 1942), thus changes to the epigenome, even in the later stages of organogenesis, may further program the embryo, and thus investigation into maintenance methylation is warranted. DNMT1 is a maintenance methyltransferase that identifies regions of hemi-methylated DNA to maintain methylation levels following replication (Hermann et al., 2004). We report an interaction in the mRNA expression of *DNMT1* where the expression level did not change in the High treated cells; however, there was a cubic response in the expression in the Low treated cells where *DNMT1* expression was equal between the Low and High ×2.5 but greater in the Low treated cells in all other EM-supplemented groups within the Low treatment. These data suggest that the increased expression in the Low Control, ×5, and ×10, compared with High-treated cells, would indicate a greater level of maintenance methylation in these treatments compared with the High glucose counterparts. DNA methylation is affected by the presence or absence of metabolites and cofactors necessary for the proper function of the one-carbon metabolism cycle. Data in multiple species has shown that mutation to or deletion of enzymes in the one-carbon metabolism cycle alters embryonic and fetal development (Narisawa et al., 2012; Cai et al., 2021). Furthermore, reduction in epigenetic modifiers to ewes altered the DNA methylation in their lambs at day 90 of gestation, where 88% of differentially methylated loci were hypomethylated or unmethylated (Sinclair et al., 2007). Embryos collected from cows supplemented with methionine or methionine and choline had decreased relative fluorescence for 5-methylcytosine compared to embryos from unsupplemented cows (0.53 and 0.62 *vs*. 0.69; Acosta et al., 2016). When comparing differentially methylated cytosines between High and Low glucose cells within the EM supplementation groups, we found that fewer cytosines were methylated in High cells, except for the comparison between High and Low ×2.5. It is worth mentioning that the expression of *DNMT1* was greater in all Low treatments, except for ×2.5 EM supplementation level. These data suggest that the increased *DNMT1* expression in Low treated cells may have resulted in increased DNMT1 abundance and activity, which would maintain greater levels of methylation compared with the High treated cells. Although measurement of the activity of DNMT1 and other methylation enzymes would have contributed to the analysis and interpretation, the combined analysis of mRNA expression and RRBS does allow for the determination of pathway directionality. While this correlation cannot be directly confirmed, it does allow for increased understanding and provides a basis for additional targeted analysis to be conducted in replicated studies *in vivo*.

It should be noted that imbalances in EM, such as that of high folate, may result in hypomethylation (Sable et al., 2015). This may be the case in our cells in which High ×5, compared with all other treatments, showed a greater number of hypomethylated cytosines, although in the case of this study High ×5 had greater growth rates and mitochondrial respiration rates than the ×2.5 and Control treatments. It would be interesting to determine the methylation level of the ×10 supplemented group to determine if a difference in methylation and methylation of particular genes could explain the difference seen in growth rates and mitochondrial respiration. Additionally, because all treatments were supplemented together and not individually, we are unable to determine whether high folate levels alone would result in a similar hypomethylation. Identifying the most beneficial EM supplement concentrations without inducing toxicity or imbalance when at specific energy balances would be crucial to the translation of this model *in vivo* to avoid aberrant methyl metabolism due to a micronutrient imbalance. Although not an area investigated with this study, global hypomethylation is a marker of most types of cancer (Videtic Paska and Hudler, 2015). The authors are currently unaware of any body of literature in the bovine to suggest an increased or decreased risk of tumorigenesis due to EM supplementation or deficiency. This may be due to B vitamin and methionine synthesis by rumen microbes, thus decreasing the risk of methyl deficiency in ruminants. For the purposes of production agriculture, the utilization of EM in cattle would be targeted to specific timepoints in the production cycle, such as during lactation and breeding (first trimester of pregnancy), in the spring when there is a greater chance of scarcity of nutrients due to drought, during which EM supplementation may be beneficial to improve lactation and programmed development.

MTR expression was affected by EM supplementation, where ×2.5 and ×5 were less than Control and ×10. Increased SAM inhibits methylenetetrahydrofolate reductase, decreasing the availability of the folate cycle methyl donor 5-methyltetrahydrofolate (5-MTF) (Jencks and Mathews, 1987). As the substrate of MTR, decreased 5-MTF may result in decreased mRNA expression of *MTR*, allowing for increased synthesis of nucleotides through the folate-dependent synthesis of purines and thymidylate (Clare et al., 2019). Increases in growth rate with increased EM supplementation may support the decrease in folate-dependent remethylation of homocysteine to methionine and increase in the synthesis of nucleotides to support cell replication and growth.

Reduced representation bisulfite sequencing and pathway analysis were conducted to determine whether the changes seen in the growth rate and mitochondrial function were due to changes in methylation of genes in pathways associated with growth. Regulation of phosphate metabolic processes, positive regulation of metabolic processes, positive regulation of GTPase, and positive regulation of MAPK cascade were common pathways of interest due to roles in protein synthesis, cell division, signal transduction, cell growth and development, and oxidative phosphorylation (Ardito et al., 2017). Multiple genes were identified across biological processes with no distinct methylation patterns except for *ADCYAP1.* Differentially methylated genes, such as *MAP2K6*, *ARRDC3*, *PDGFA*, *SFRP1*, *IL2*, and *RAC1*, are involved with maintaining cell growth rates, activation of GTPases and kinases as well as responses to inflammation and environmental stimulus, all of which affected the differential growth and metabolic rates of the embryonic fibroblasts. The consistent methylation patterns identified with *ADCYAP1* may identify this gene as a target for future study. Specifically, *ADCYAP1*, a gene differentially methylated and over-represented in all biological processes, was also differentially methylated in all treatment comparisons. *ADCYAP1*decreased in methylation with increased EM supplementation level in High cells, while it increased in methylation in the Low cells. There is a single CpG island in the promoter region of *ADCYAP1*, and hypermethylation of the promoter region is responsible for transcriptional silencing (Jung et al., 2011) and thus the transcriptional repression of downstream targets. Decreased methylation of *ADCYAP1* increases transcription in response to glucose concentrations. Increased glucose results in increased cyclic AMP levels, which result in the phosphorylation cascade to increased glucose metabolism (Ravnskjaer et al., 2016). Therefore, differential methylation of *ADCYAP1* in response to glucose treatment and increased EM supplementation in the High cells may explain the increased cell growth rate and proliferation of the High cells compared with the Low as well as the increase in ×2.5, ×5, and ×10 in the High cells.

## Conclusion

These novel data presented herein add to previously published data showing the positive benefits of one or two EM and demonstrate that the further inclusion of four EM increases growth rate and mitochondrial respiration depending on cell energy availability. Furthermore, these changes in growth rate and mitochondrial respiration may be driven by differential methylation of genes involved in biological pathways related to phosphorylation and positive regulation of metabolism. Additionally, further research into how the energy status affects EM utilization and whole-body metabolism and growth rate is warranted. These data support the translation of this model to an *in vivo* system and the need to identify how the inclusion of four EM in the diet affects maternal and embryonic metabolism in cattle and humans. Lastly, evaluating the responses of EM supplementation to dams at different energy intakes is warranted to identify whether embryonic responses *in vivo* are similar to responses *in vitro*.

## Data Availability

The datasets presented in this study can be found in online repositories. The name of the repository and accession number can be found below: https://www.ncbi.nlm.nih.gov/geo/, GSE180362.
