# Supplementary material for: Epigenetic Modifier Supplementation Improves Mitochondrial Respiration and Growth Rates and Alters DNA Methylation of Bovine Embryonic Fibroblast Cells Cultured in Divergent Energy Supply
Source: Front Genet. 2022 Feb 24;13:812764. doi: 10.3389/fgene.2022.812764 (PMC8907857; doi:10.3389/fgene.2022.812764)
Supplement: Supplementary file 3 [file DataSheet1.docx]

| **Supplementary Table 1.** Primers for real-time PCR assays. | | | | | |
| --- | --- | --- | --- | --- | --- |
| Symbol | Gene Name | Assay ID | Primer Efficiency | Linearity | M Value |
| *MAT1A* | Methionine Adenosyltransferase 1A | Bt03235690_m1 | Not Expressed | - | - |
| *MAT2A* | Methionine Adenosyltransferase 2A | Bt03271823_m1 | 89.72 | 0.987 | - |
| *MAT2B* | Methionine Adenosyltransferase 2B | Bt03240156_g1 | 87.61 | 0.994 | - |
| *DNMT1* | DNA Methyltransferase 1 | Bt03224754_m1 | 89.82 | 0.998 | - |
| *DNMT3A* | DNA Methyltransferase 3A | Bt01027164_m1 | 96.80 | 0.951 | - |
| *DNMT3B* | DNA Methyltransferase 3B | Bt03259818_m1 | 91.80 | 0.916 | - |
| *AHCY* | S-Adenosylhomocysteine Hydrolase | Bt03225928_m1 | 94.71 | 0.994 | - |
| *MTR* | Methionine Synthase | Bt03220598_m1 | 89.84 | 0.987 | - |
| *BHMT* | Betaine-Homocysteine S- Methyltransferase | Bt03210343_m1 | Not Expressed | - | - |
| *ACTB* | β-Actin | Bt03279174_g1 | 85.42 | 0.997 | 0.279 |
| *GAPDH* | Glyceraldehyde-3-Phosphate Dehydrogenase | Bt03210913_g1 | 89.05 | 0.996 | 0.297 |
| *SDHA* | Succinate Dehydrogenase | Bt04307498_m1 | 89.80 | 0.996 | 0.254 |

| **Supplementary Table 2.** Growth rate of Embryonic Tracheal Fibroblast (EBTr) cells as influenced by glucose and epigenetic modifiers (EM) level in Eagle’s Minimum Essential Medium (EMEM). | | | | | | | | | | | | | |
| --- | --- | --- | --- | --- | --- | --- | --- | --- | --- | --- | --- | --- | --- |
|  |  | Epigenetic Modifiers^1^ | | | |  |  | *P*-value^2^ | | | | | |
| Time, h^3^ | Glucose^4^ | CON | 2.5X | 5X | 10X | Average Glc^5^ | SEM^6^ | Glc | EM | Glc × EM | Linear | Quad | Cubic |
| 12 | Low | 186.0 | 153.4 | 204.1 | 150.5 | 173.5^b^ | 16.2 | < 0.01 | 0.03 | 0.08 | 0.10 | 0.13 | < 0.01 |
|  | High | 219.2 | 199.2 | 220.4 | 229.9 | 217.2^a^ |  |  |  |  | 0.35 | 0.47 | 0.30 |
|  | EMAvg^7^ | 202.6^c^ | 176.3^cd^ | 212.2^c^ | 190.2^cd^ |  |  |  |  |  |  |  |  |
| 24 | Low | 257.5^gh^ | 211.3^i^ | 269.7^fg^ | 224.9^hi^ | 240.9 | 17.2 | < 0.01 | 0.12 | 0.01 | 0.21 | 0.51 | < 0.01 |
|  | High | 301.1^e^ | 298.8^ef^ | 296.7^ef^ | 332.9^e^ | 307.4 |  |  |  |  | 0.13 | 0.30 | 0.80 |
|  | EM Avg | 279.3 | 255.1 | 283.2 | 278.9 |  |  |  |  |  |  |  |  |
| 36 | Low | 324.5^h^ | 311.8^h^ | 416.9^g^ | 337.0^h^ | 347.5 | 22.1 | < 0.01 | < 0.01 | < 0.01 | 0.04 | < 0.01 | < 0.01 |
|  | High | 453.5^g^ | 534.0^f^ | 519.3^f^ | 595.6^e^ | 525.6 |  |  |  |  | < 0.01 | 0.60 | 0.11 |
|  | EM Avg | 389.0 | 422.9 | 468.1 | 466.3 |  |  |  |  |  |  |  |  |
| 48 | Low | 489.1^g^ | 458.2^g^ | 591.3^ef^ | 347.1^h^ | 471.3 | 43.4 | < 0.01 | 0.05 | < 0.01 | < 0.01 | < 0.01 | 0.01 |
|  | High | 524.6^fg^ | 658.7^e^ | 552.1^fg^ | 632.9^ef^ | 592.1 |  |  |  |  | 0.18 | 0.63 | 0.02 |
|  | EM Avg | 506.8 | 558.5 | 571.7 | 490.0 |  |  |  |  |  |  |  |  |
| 72 | Low | 707.8^h^ | 641.4^h^ | 1020.7^g^ | 636.3^h^ | 751.6 | 56.5 | < 0.01 | < 0.01 | < 0.01 | 0.79 | < 0.01 | < 0.01 |
|  | High | 1029.1^g^ | 1256.1^f^ | 1227.6^f^ | 1620.6^e^ | 1283.4 |  |  |  |  | < 0.01 | 0.38 | 0.02 |
|  | EM Avg | 868.5 | 948.7 | 1124.2 | 1128.5 |  |  |  |  |  |  |  |  |
| ^1^CON = Basal concentrations of Methionine, Folate, and Choline in EMEM media with 4 µmol/L Vitamin B_12_. 2.5X, 5X, 10X = 2.5, 5, and 10 times the concentration of Folate, Choline, and Vitamin B_12_ in CON media. Methionine limited to 2X CON.  ^2^Probability values for the effects of glucose, EM, and the interaction. Linear, quadratic, and cubic polynomial contrasts for the effect of glucose and increasing EM supplementation on growth rate of EBTr cells within glucose level.  ^3^Hours after plating.  ^4^Low = 1 g/L glucose and High = 4.5 g/L glucose in culture media.  ^5^Average growth rate within glucose level.  ^6^Standard error of the mean for the interaction of glucose and EM level.  ^7^Average growth rate within EM supplementation level.  ^a-b^Means within a column without a common superscript differ. Only presented in the lack of the Glc × EM interaction.  ^c-d^Means within a EM without a common superscript differ. Only presented in the lack of the Glc × EM interaction.  ^e-i^Means within an hour without a common superscript differ. | | | | | | | | | | | | | |

| **Supplementary Table 3.** Labeling index (cell proliferation) of Embryonic Tracheal Fibroblast (EBTr) cells stained with Ki67 as influenced by glucose and epigenetic modifier (EM) level in Eagle’s Minimum Essential Medium (EMEM)^1^. | | | | | | | | | | | | | |
| --- | --- | --- | --- | --- | --- | --- | --- | --- | --- | --- | --- | --- | --- |
|  |  | One-Carbon Metabolites^2^ | | | |  |  | *P*-value^3^ | | | | | |
| Time, h^4^ | Glucose^5^ | CON | 2.5X | 5X | 10X | Average Glc^6^ | SEM^7^ | Glc | EM | Glc × EM | Linear | Quad | Cubic |
| 1 | Low | 30.67^a^ | 32.13^a^ | 32.28^a^ | 37.86^b^ | 33.23 | 1.85 | 0.44 | 0.77 | 0.05 | 0.50 | 0.14 | 0.27 |
|  | High | 32.59^ab^ | 32.50^ab^ | 35.73^ab^ | 30.60^a^ | 32.85 |  |  |  |  | 0.03 | 0.50 | 0.67 |
|  | EM Avg^8^ | 31.63 | 32.32 | 34.00 | 34.23 |  |  |  |  |  |  |  |  |
| 12 | Low | 9.73 | 11.69 | 12.57 | 12.25 | 11.56 | 2.01 | 0.19 | 0.60 | 0.65 | 0.14 | 0.19 | 0.91 |
|  | High | 10.35 | 13.91 | 13.28 | 10.83 | 12.09 |  |  |  |  | 0.87 | 0.14 | 0.55 |
|  | EM Avg | 10.04 | 12.80 | 12.93 | 11.54 |  |  |  |  |  |  |  |  |
| 24 | Low | 13.46^ab^ | 15.34^ab^ | 14.63^ab^ | 12.46^a^ | 13.97 | 1.40 | 0.03 | 0.07 | 0.02 | 0.25 | 0.09 | 0.49 |
|  | High | 12.85^a^ | 16.40^b^ | 15.03^ab^ | 18.41^c^ | 15.67 |  |  |  |  | 0.01 | 0.78 | 0.10 |
|  | EM Avg | 13.15 | 13.87 | 14.83 | 15.44 |  |  |  |  |  |  |  |  |
| 36 | Low | 10.56^bc^ | 11.55^bc^ | 10.32^bc^ | 8.49^a^ | 10.23 | 0.77 | 0.75 | 0.06 | 0.02 | 0.01 | 0.10 | 0.24 |
|  | High | 10.14^bc^ | 10.50^c^ | 9.02^ab^ | 10.75^c^ | 10.10 |  |  |  |  | 0.62 | 0.15 | 0.11 |
|  | EM Avg | 10.35 | 11.02 | 9.67 | 9.62 |  |  |  |  |  |  |  |  |
| 48 | Low | 8.88^ab^ | 7.39^a^ | 9.27^ab^ | 7.98^a^ | 8.38 | 1.02 | <0.01 | 0.56 | 0.03 | 0.71 | 0.86 | 0.07 |
|  | High | 10.43^bc^ | 12.21^cd^ | 11.00^bc^ | 13.25^d^ | 11.72 |  |  |  |  | 0.05 | 0.80 | 0.11 |
|  | EM Avg | 9.65 | 9.80 | 10.13 | 10.62 |  |  |  |  |  |  |  |  |
| 72 | Low | 5.59^d^ | 4.53^abc^ | 4.99^bcd^ | 3.87^a^ | 4.74 | 0.48 | 0.74 | 0.83 | 0.01 | 0.04 | 0.94 | 0.19 |
|  | High | 4.36^ab^ | 4.78^abcd^ | 4.63^abcd^ | 5.41^cd^ | 4.80 |  |  |  |  | <0.01 | 0.56 | 0.19 |
|  | EM Avg | 4.97 | 4.66 | 4.81 | 4.64 |  |  |  |  |  |  |  |  |
| ^1^Labeling index = % of positively stained cells with Ki67.  ^2^CON = Basal concentrations of Methionine, Folate, and Choline in EMEM media with 4 µmol/L Vitamin B_12_. 2.5X, 5X, 10X = 2.5, 5, and 10 times the concentration of Folate, Choline, and Vitamin B_12_ in CON media. Methionine limited to 2X CON.  3Probability values for the effects of glucose, EM, and the interaction. Linear, quadratic (Quad), and cubic polynomial contrasts for the effect of glucose and increasing EM on growth rate of EBTr cells within glucose level.  ^4^Hours after plating.  ^5^Low = 1 g/L glucose and High = 4.5 g/L glucose in culture media.  ^6^Average growth rate within glucose level.  ^7^Standard error of the mean for the interaction of glucose and EM level.  ^8^Average growth rate within EM supplementation level.  ^a-d^Means within an hour without a common superscript differ. | | | | | | | | | | | | | |

| **Supplementary Table 4.** Mitochondrial respiration parameters of Embryonic Tracheal Fibroblast (EBTr) cells as influenced by glucose and epigenetic modifiers (EM) level in Eagle’s Minimum Essential Medium (EMEM).^1^ | | | | | | | | | | | | | |
| --- | --- | --- | --- | --- | --- | --- | --- | --- | --- | --- | --- | --- | --- |
|  |  | EM Concentrations^2^ | | | |  |  | *P* – values^3^ | | | | | |
| Meas^4^ | Glc^5^ | CON | 2.5X | 5X | 10X | Avg Glc^6^ | SEM | Glc | EMM | Glc × EM | Linear | Quad | Cubic |
| Baseline | Low | 26.2^a^ | 34.0^c^ | 26.7^a^ | 26.6^a^ | 28.1 | 0.52 | 0.01 | 0.01 | 0.01 | 0.01 | 0.01 | 0.01 |
|  | High | 28.5^b^ | 38.7^d^ | 34.1^c^ | 37.9^d^ | 34.8 |  |  |  |  | 0.01 | 0.01 | 0.01 |
|  | EM Avg^7^ | 27.3 | 36.4 | 30.4 | 31.8 |  |  |  |  |  |  |  |  |
| O_2_-linked ATP | Low | 16.3^a^ | 21.6^b^ | 16.3^a^ | 14.5^a^ | 17.2 | 0.61 | 0.01 | 0.01 | 0.01 | 0.01 | 0.01 | 0.01 |
|  | High | 20.3^b^ | 24.0^c^ | 23.4^c^ | 24.1^c^ | 22.9 |  |  |  |  | 0.01 | 0.02 | 0.04 |
|  | EM Avg | 18.2 | 22.8 | 19.8 | 19.3 |  |  |  |  |  |  |  |  |
| Maximal Resp | Low | 43.0 | 53.0 | 48.8 | 36.5 | 45.3 | 2.65 | 0.01 | 0.01 | 0.01 | 0.05 | 0.01 | 0.26 |
|  | High | 43.0 | 64.7 | 51.2 | 65.5 | 56.1 |  |  |  |  | 0.01 | 0.19 | 0.01 |
|  | EM Avg | 43.0 | 58.8 | 50.0 | 51.0 |  |  |  |  |  |  |  |  |
| Reserve capacity | Low | 16.9^abc^ | 19.0^bc^ | 22.1^cd^ | 10.8^a^ | 17.2 | 2.25 | 0.02 | 0.06 | 0.01 | 0.11 | 0.04 | 0.47 |
|  | High | 14.5^ab^ | 26.0^d^ | 17.0^abc^ | 27.6^d^ | 21.3 |  |  |  |  | 0.01 | 0.99 | 0.01 |
|  | EM Avg | 15.7 | 22.5 | 19.6 | 19.2 |  |  |  |  |  |  |  |  |
| Proton leak | Low | 3.3 | 3.5 | 5.1 | 4.5 | 4.1 | 0.75 | 0.06 | 0.44 | 0.48 | 0.35 | 0.47 | 0.46 |
|  | High | 3.5 | 2.3 | 3.0 | 3.3 | 3.0 |  |  |  |  | 0.85 | 0.07 | 0.07 |
|  | EM Avg | 3.4 | 2.9 | 4.0 | 3.9 |  |  |  |  |  |  |  |  |
| Non-mito resp. | Low | 6.6^b^ | 8.9^d^ | 5.3^a^ | 6.7^bc^ | 6.9 | 0.38 | 0.01 | 0.01 | 0.01 | 0.13 | 0.76 | 0.01 |
|  | High | 4.9^a^ | 12.5^f^ | 7.8^cd^ | 10.5^e^ | 8.9 |  |  |  |  | 0.01 | 0.01 | 0.01 |
|  | EM Avg | 5.8 | 10.7 | 6.5 | 8.6 |  |  |  |  |  |  |  |  |
| ^1^O_2_ consumption pmol/min/cells scaled to 10,000 cells  ^2^CON = Basal concentrations of Methionine, Folate, and Choline in EMEM media with 4 µmol/L Vitamin B_12_. 2.5X, 5X, 10X = 2.5, 5, and 10 times the concentration of Folate, Choline, and Vitamin B_12_ in CON media. Methionine limited to 2X CON.  ^3^Probability values for the effect of glucose, epigenetic modifier, and the interaction. Polynomial contrasts within glucose level across EM include linear, quadratic, and cubic.  ^4^Measurement: Baseline = Basal cellular respiration. O2-linked ATP synthesis = baseline O_2_ consumption minus Oligomycin. Reserve capacity = maximal respiration minus baseline. Proton leak = oligomycin O_2_ minus non-mitochondrial respiration. Non-mitochondrial respiration (Non-mito resp.) = O_2_ consumption after rotenone/antimycin A.  ^5^Glucose concentrations in EMEM media. Low = 1 g/L. High = 4.5 g/L.  ^6^Average mitochondrial respiration parameter within a glucose level.  ^7^Average mitochondrial respiration parameter within an EM level.  ^a-f^Means within a measurement without common superscript within row and column differ. | | | | | | | | | | | | | |

| **Supplementary Table 5.** Growth rate of Embryonic Tracheal Fibroblast (EBTr) cells as influenced by glucose and epigenetic modifiers (EM) level in Eagle’s Minimum Essential Medium (EMEM). | | | | | | | | | | | | | |
| --- | --- | --- | --- | --- | --- | --- | --- | --- | --- | --- | --- | --- | --- |
|  |  | Epigenetic Modifiers^1^ | | | |  |  | *P*-value^2^ | | | | | |
| Gene^3^ | Glucose^4^ | CON | 2.5X | 5X | 10X | Average Glc^5^ | SEM^6^ | Glc | EM | Glc × EM | Linear | Quad | Cubic |
| *MAT2A* | Low | 1.36 | 1.27 | 1.03 | 1.02 | 1.17^a^ | 0.08 | 0.02 | 0.09 | 0.11 | 0.02 | 0.28 | 0.38 |
|  | High | 1.00 | 1.07 | 0.98 | 1.05 | 1.03^b^ |  |  |  |  | 0.76 | 0.85 | 0.25 |
|  | EMAvg^7^ | 1.18 | 1.17 | 1.00 | 1.04 |  |  |  |  |  |  |  |  |
| *MAT2B* | Low | 1.06^d^ | 0.80^c^ | 0.89^cd^ | 0.94^cd^ | 0.92 | 0.06 | < 0.01 | 0.02 | < 0.01 | 0.43 | 0.01 | 0.03 |
|  | High | 1.00^d^ | 1.39^e^ | 1.52^e^ | 1.55^e^ | 1.36 |  |  |  |  | 0.01 | 0.02 | 0.53 |
|  | EM Avg | 1.03 | 1.09 | 1.20 | 1.25 |  |  |  |  |  |  |  |  |
| *DNMT1* | Low | 1.34^d^ | 0.80^d^ | 1.24^d^ | 1.38^d^ | 1.19 | 0.08 | < 0.01 | < 0.01 | 0.01 | 0.06 | 0.01 | 0.01 |
|  | High | 1.00^c^ | 0.90^c^ | 1.01^c^ | 0.91^c^ | 0.96 |  |  |  |  | 0.64 | 0.92 | 0.29 |
|  | EM Avg | 1.17 | 0.85 | 1.13 | 1.15 |  |  |  |  |  |  |  |  |
| *DNMT3A* | Low | 1.09 | 0.81 | 0.92 | 0.99 | 0.95 | 0.08 | 0.36 | 0.09 | 0.52 | 0.84 | 0.14 | 0.22 |
|  | High | 1.00 | 0.90 | 1.00 | 1.13 | 1.01 |  |  |  |  | 0.09 | 0.24 | 0.37 |
|  | EM Avg | 1.05 | 0.86 | 0.96 | 1.06 |  |  |  |  |  |  |  |  |
| *DNMT3B* | Low | 0.73 | 0.53 | 1.00 | 1.00 | 0.81 | 0.12 | 0.41 | 0.07 | 0.20 | 0.01 | 0.98 | 0.01 |
|  | High | 1.00 | 0.77 | 0.81 | 0.96 | 0.89 |  |  |  |  | 0.94 | 0.26 | 0.65 |
|  | EM Avg | 0.86 | 0.65 | 0.90 | 0.98 |  |  |  |  |  |  |  |  |
| *AHCY* | Low | 0.57 | 0.73 | 0.66 | 0.68 | 0.66^b^ | 0.08 | < 0.01 | 0.78 | 0.15 | 0.35 | 0.21 | 0.11 |
|  | High | 1.00 | 0.78 | 0.79 | 0.93 | 0.87^a^ |  |  |  |  | 0.90 | 0.13 | 0.63 |
|  | EM Avg | 0.78 | 0.76 | 0.72 | 0.80 |  |  |  |  |  |  |  |  |
| *MTR* | Low | 1.27 | 0.92 | 0.94 | 1.13 | 1.04 | 0.10 | 0.33 | 0.03 | 0.31 | 0.56 | 0.01 | 0.70 |
|  | High | 1.00 | 0.85 | 0.94 | 1.09 | 0.97 |  |  |  |  | 0.35 | 0.28 | 0.45 |
|  | EM Avg | 1.13^f^ | 0.88^g^ | 0.89^g^ | 1.11^f^ |  |  |  |  |  |  |  |  |
| ^1^CON = Basal concentrations of Methionine, Folate, and Choline in EMEM media with 4 µmol/L Vitamin B_12_. 2.5X, 5X, 10X = 2.5, 5, and 10 times the concentration of Folate, Choline, and Vitamin B_12_ in CON media. Methionine limited to 2X CON.  ^2^Probability values for the effects of glucose, EM, and the interaction. Linear, quadratic, and cubic polynomial contrasts for the effect of glucose and increasing EM supplementation on growth rate of EBTr cells within glucose level.  ^3^*MAT2A* and *2B*: Methionine Adenosyltransferase 2A and 2B. *DNMT1*, 3A, and 3B: DNA Methyltransferase 1, 3A, and 3B. *AHCY*: S-Adenosylhomocysteine Hydrolase, and *MTR*: Methionine Synthase.  ^4^Low = 1 g/L glucose and High = 4.5 g/L glucose in culture media.  ^5^Average mRNA expression within glucose level.  ^6^Standard error of the mean for the interaction of glucose and EM level.  ^7^Average mRNA expression within EM supplementation level.  ^a-b^Means within a column without a common superscript differ. Only presented in the lack of the Glc × EM interaction.  ^f-g^Means within a EM without a common superscript differ. Only presented in the lack of the Glc × EM interaction.  ^c-e^Means within a gene without a common superscript differ. | | | | | | | | | | | | | |
