# Supplementary material for: Epigenetic Modifier Supplementation Improves Mitochondrial Respiration and Growth Rates and Alters DNA Methylation of Bovine Embryonic Fibroblast Cells Cultured in Divergent Energy Supply
Source: Front Genet. 2022 Feb 24;13:812764. doi: 10.3389/fgene.2022.812764 (PMC8907857; doi:10.3389/fgene.2022.812764)

Polynomial Contrasts:

The difference in statistical analysis between an ANOVA and the polynomial contrast is that the polynomial contrast combines all 4 levels of EM within a glucose level into one analysis and measures for linear, quadratic, or cubic increases/decreases in means with increasing supplementation level within a glucose treatment. By doing this measurement, it increases the power of the test compared with the MIXED model which evaluates each glucose and EM treatment mean independently. Because there are 4 treatments within a glucose level, there can be three contrasts run, linear, quadratic, and cubic. These contrasts are conducted as a linear, quadratic, or cubic response from Control to 10X. In the graphs below, the highest order statistically significant contrast is presented. Low contrasts are solid black lines. High contrasts are the dashed lines. Only measurements with significant polynomial contrasts are presented for visualization, and only highest order significant polynomial contrasts are visualized.

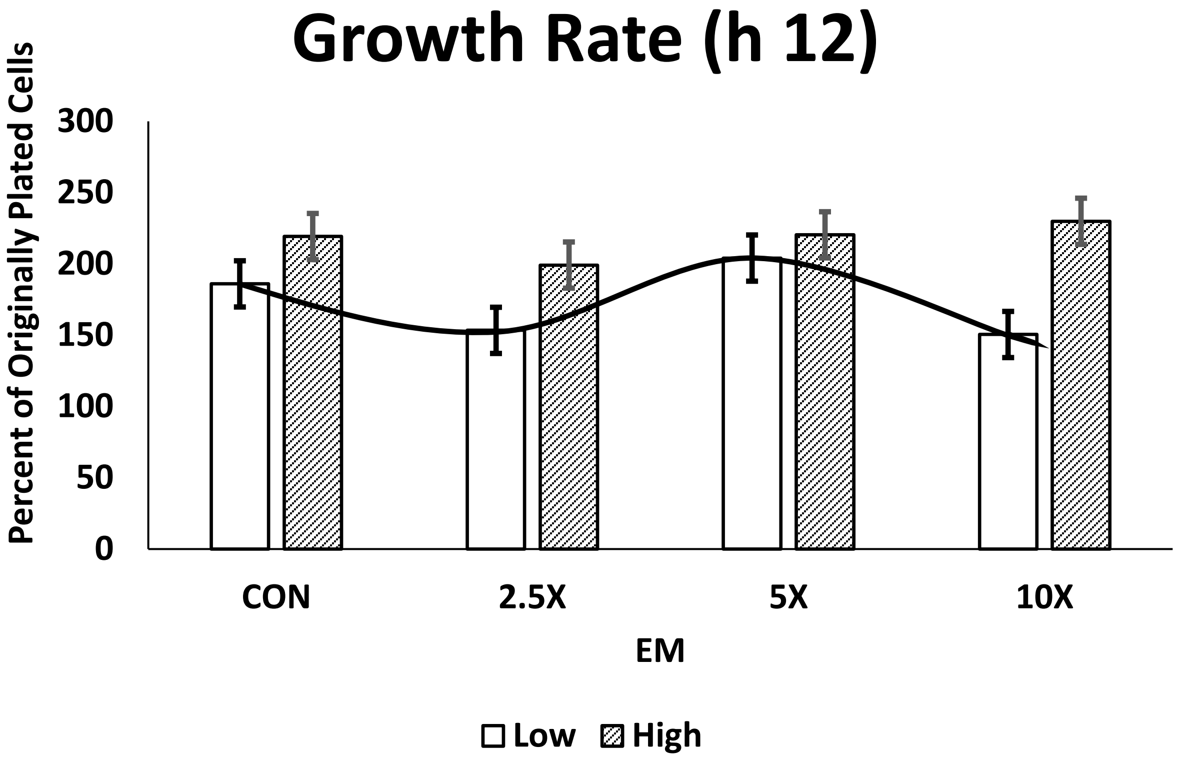

Growth Rate (h 12)
Percent of Originally Plated Cells
300
250
200
150
100
50
0
CON
2.5X
5X
10X
EM
Low
High

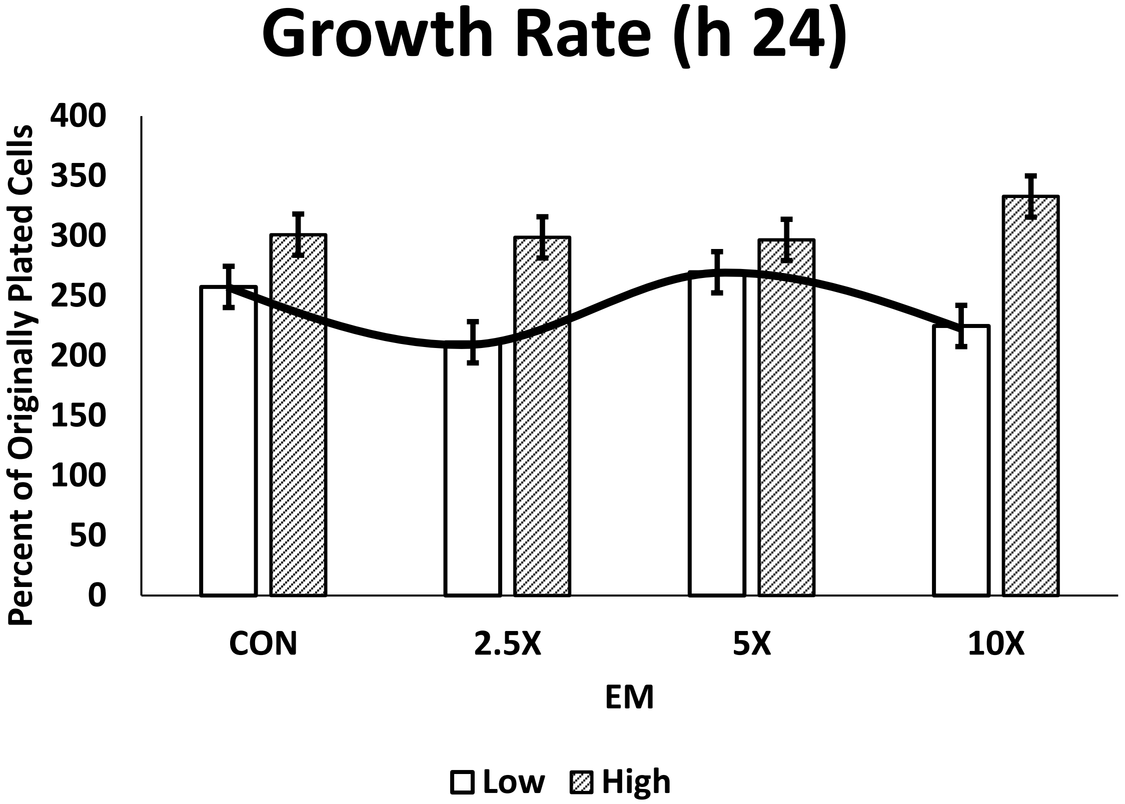

Growth Rate (h 24)
Percent of Originally Plated Cells
400
350
300
250
200
150
100
50
0
CON
2.5X
5X
10X
EM
Low
High

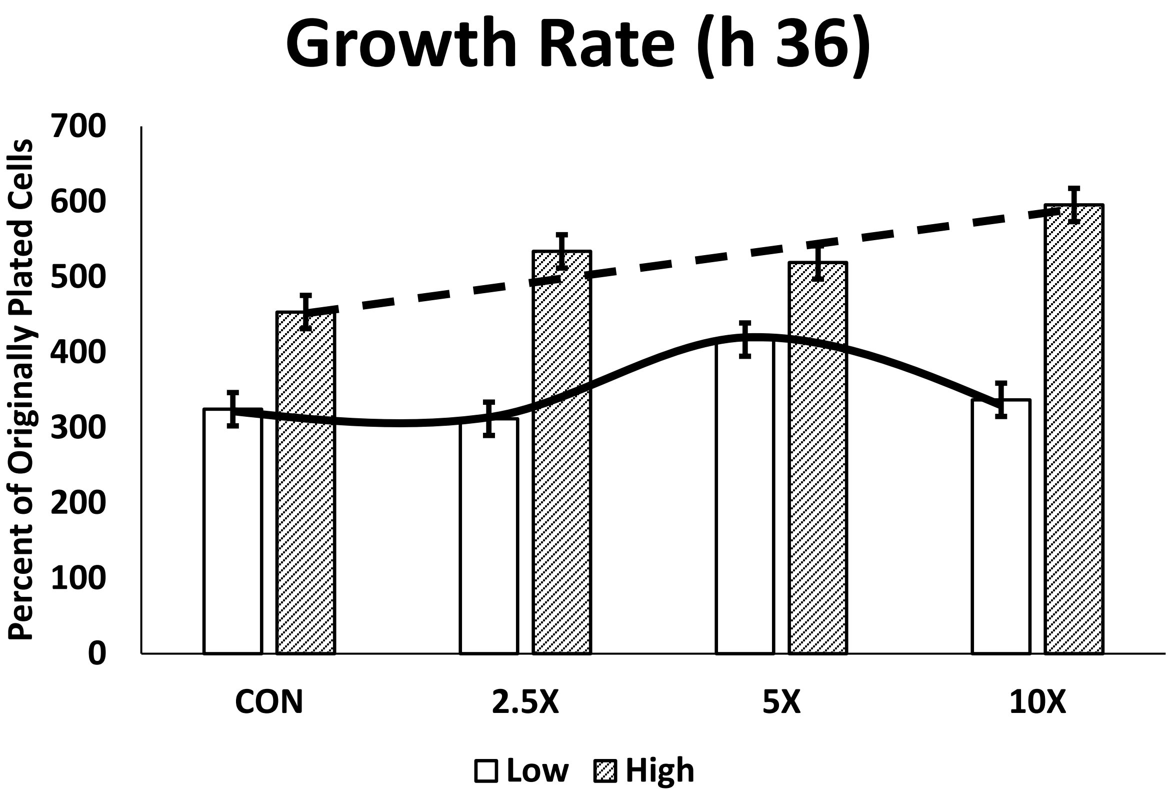
Growth Rate (h 36)
Percent of Originally Plated Cells
700
600
500
400
300
200
100
0
CON
2.5X
5X
10X
Low
High

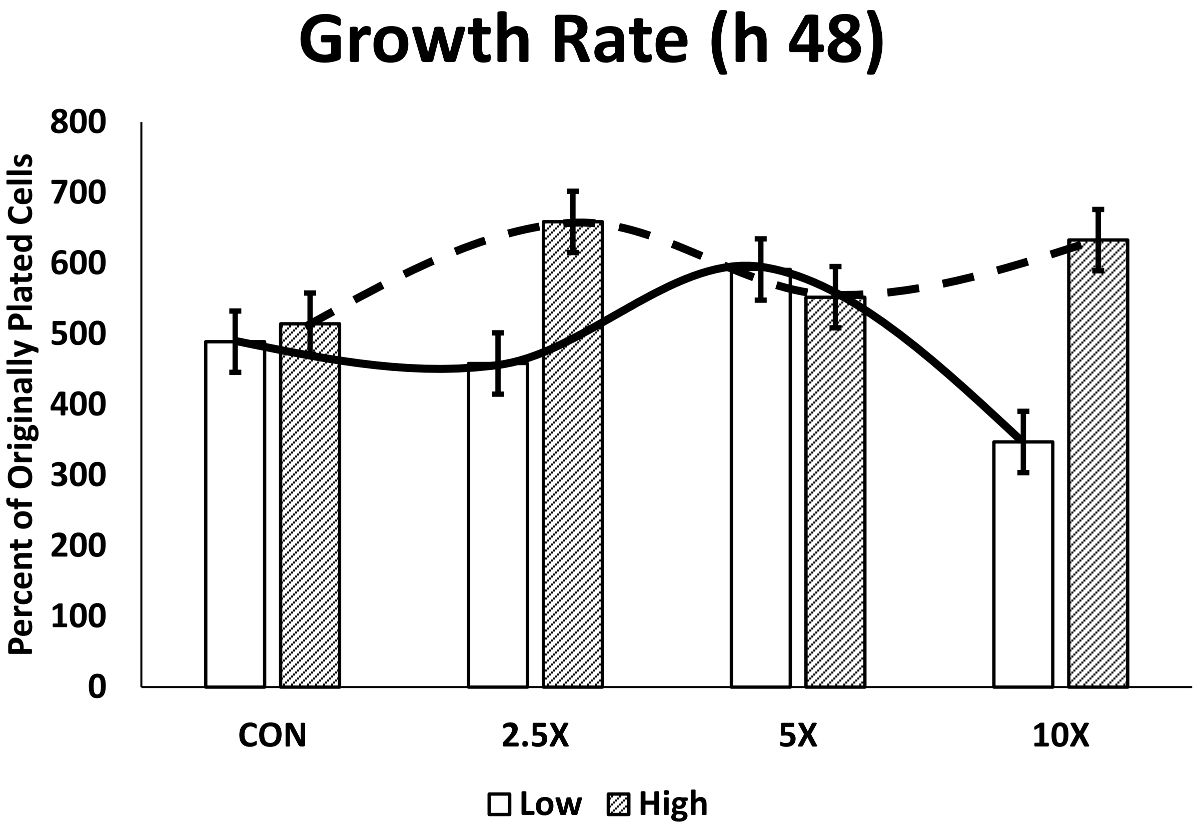
Growth Rate (h 48)
Percent of Originally Plated Cells
800
700
600
500
400
300
200
100
0
CON
2.5X
5X
10X
Low
High

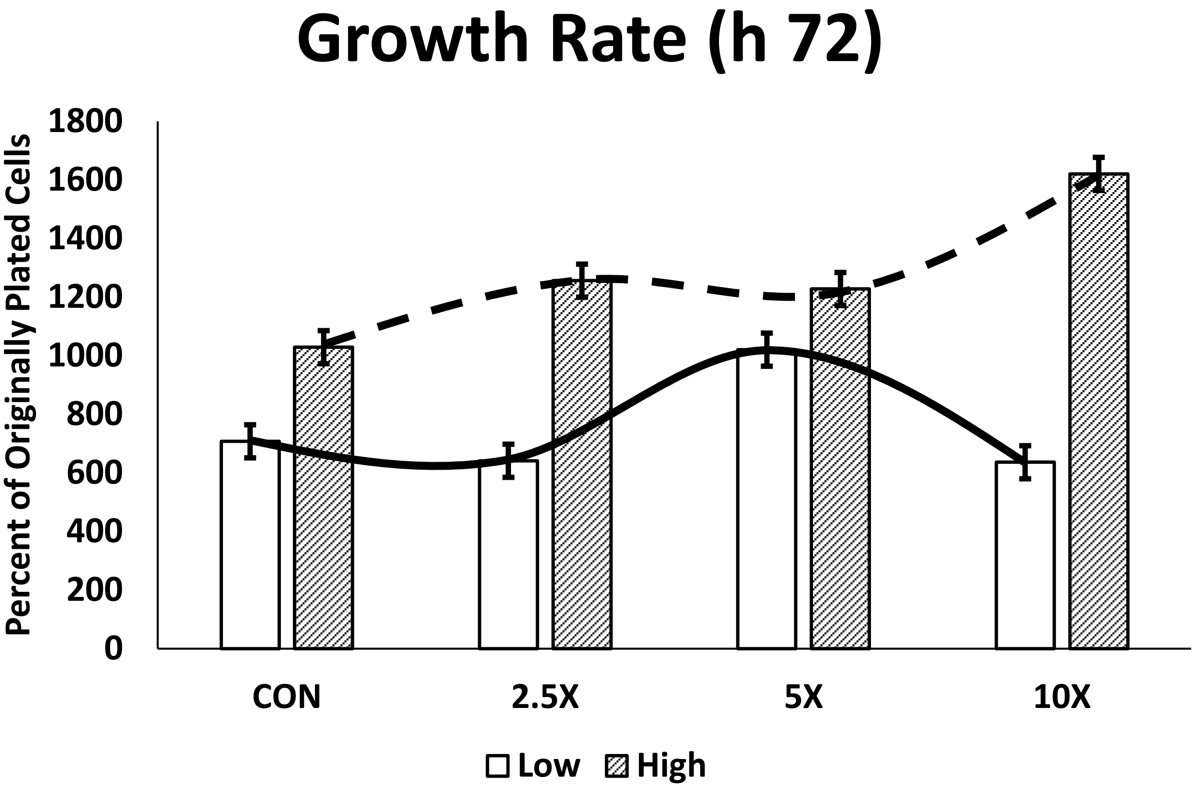
Growth Rate (h 72)
Percent of Originally Plated Cells
1800
1600
1400
1200
1000
800
600
400
200
0
CON
2.5X
5X
10X
Low
High

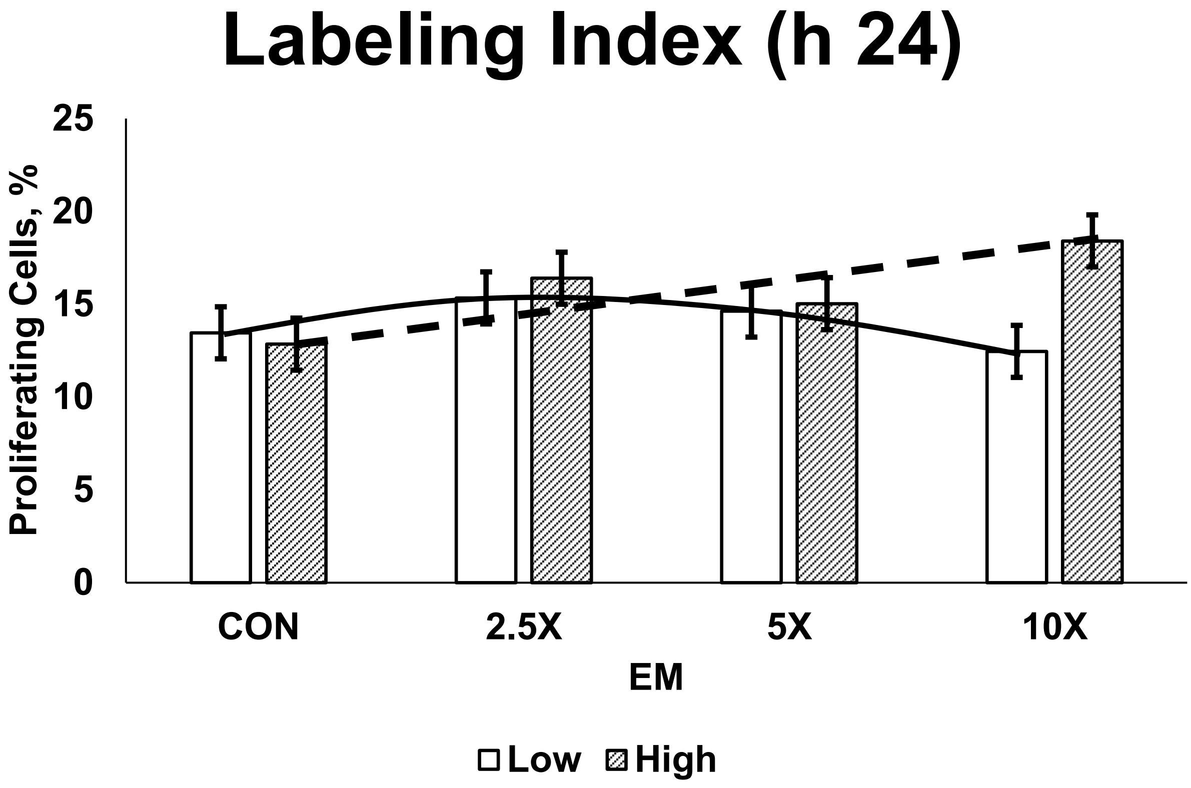
Labeling Index (h 24)
Proliferating Cells, %
25
20
15
10
5
0
CON
2.5X
5X
10X
EM
Low
High

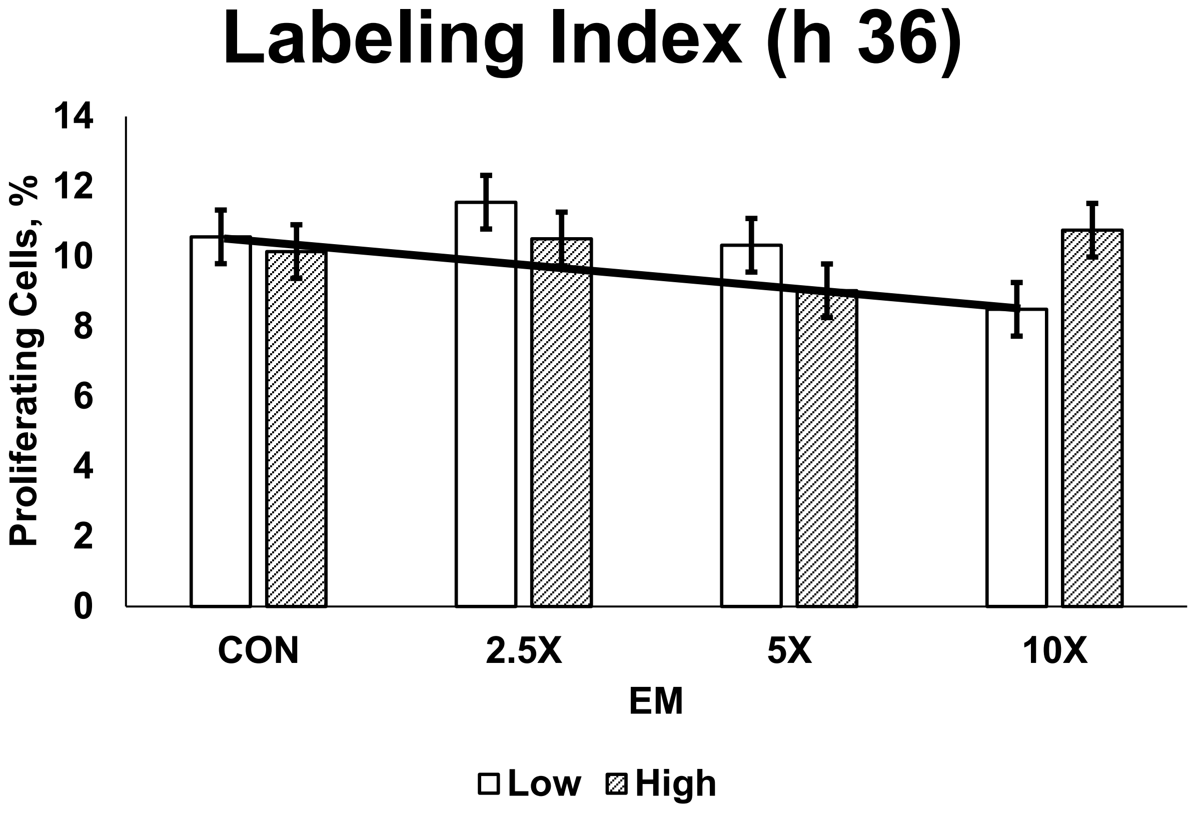
Labeling Index (h 36)
Proliferating Cells, %
14
12
10
8
6
4
2
0
CON
2.5X
5X
10X
EM
Low
High

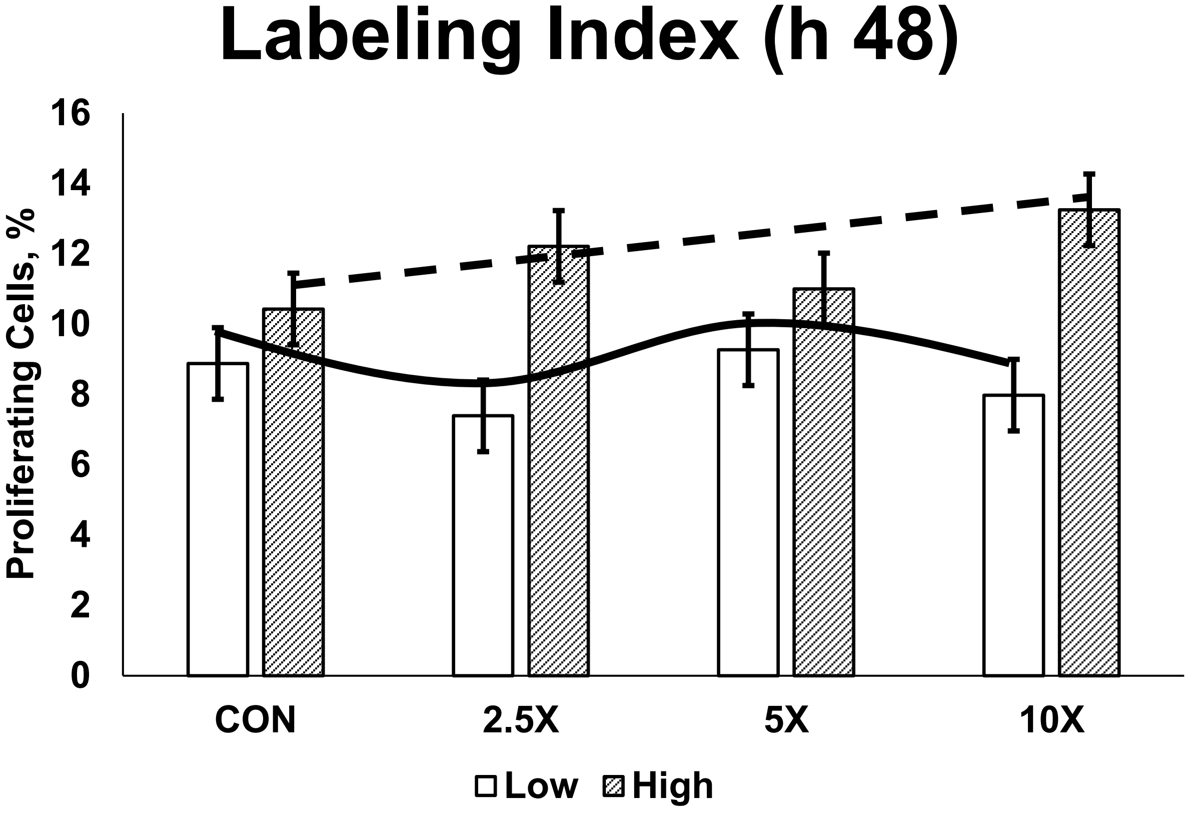

Labeling Index (h 48)
Proliferating Cells, %
16
14
12
10
8
6
4
2
0
CON
2.5X
5X
10X
Low
High

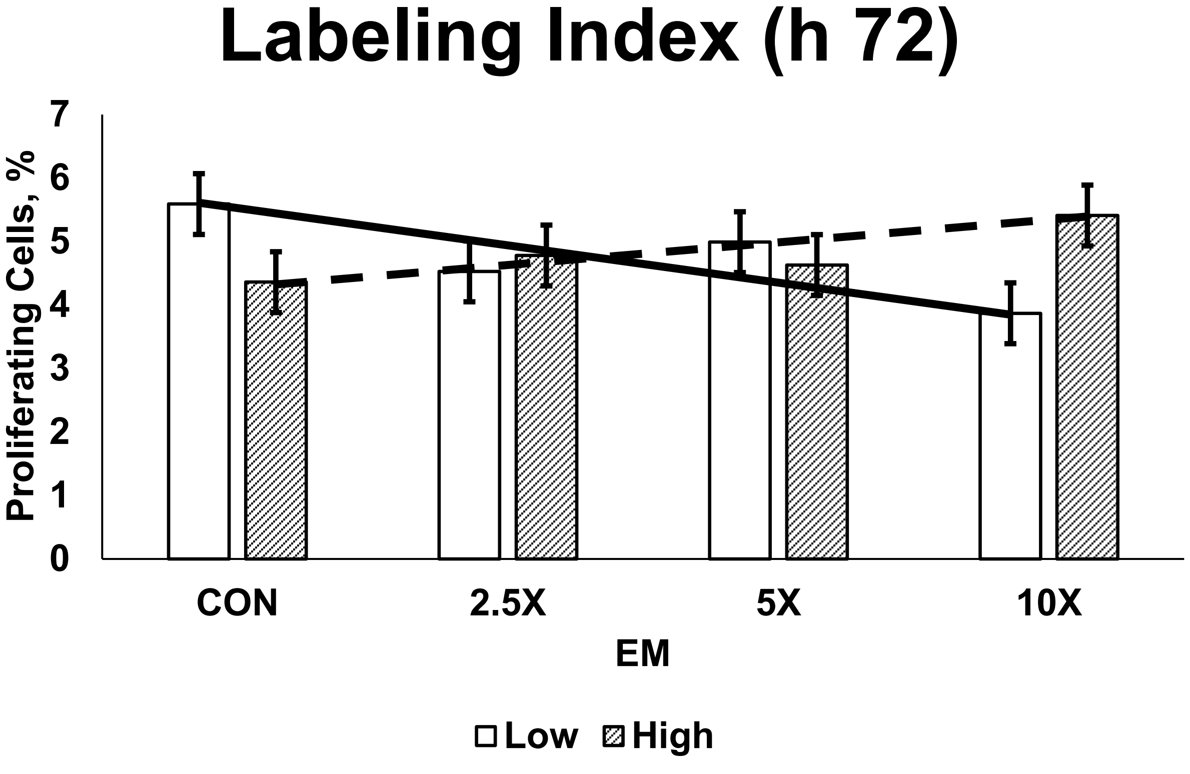

Labeling Index (h 72)
Proliferating Cells, %
7
6
5
4
3
2
1
0
CON
2.5X
5X
10X
EM
Low
High

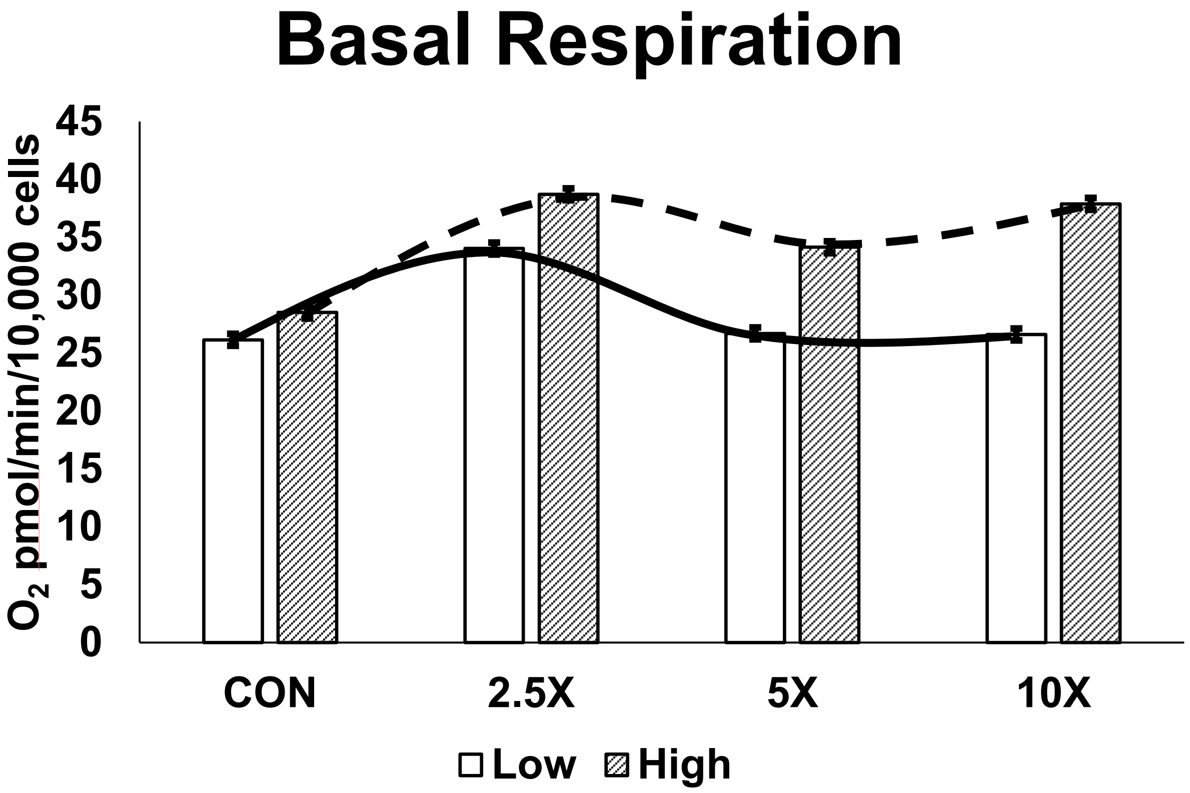
Basal Respiration
O2 pmol/min/10,000 cells
45
40
35
30
25
20
15
10
5
0
CON
2.5X
5X
10X
Low
High

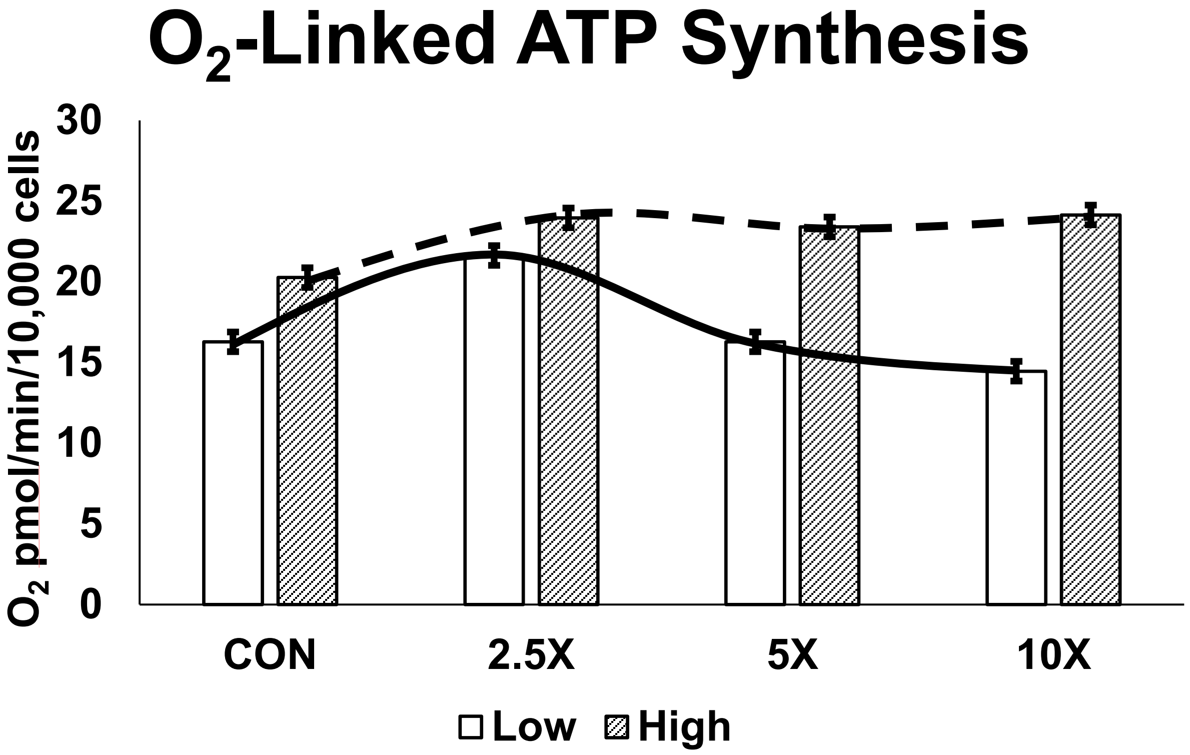
O2-Linked ATP Synthesis
O2 pmol/min/10,000 cells
30
25
20
15
10
5
0
CON
2.5X
5X
10X
Low
High

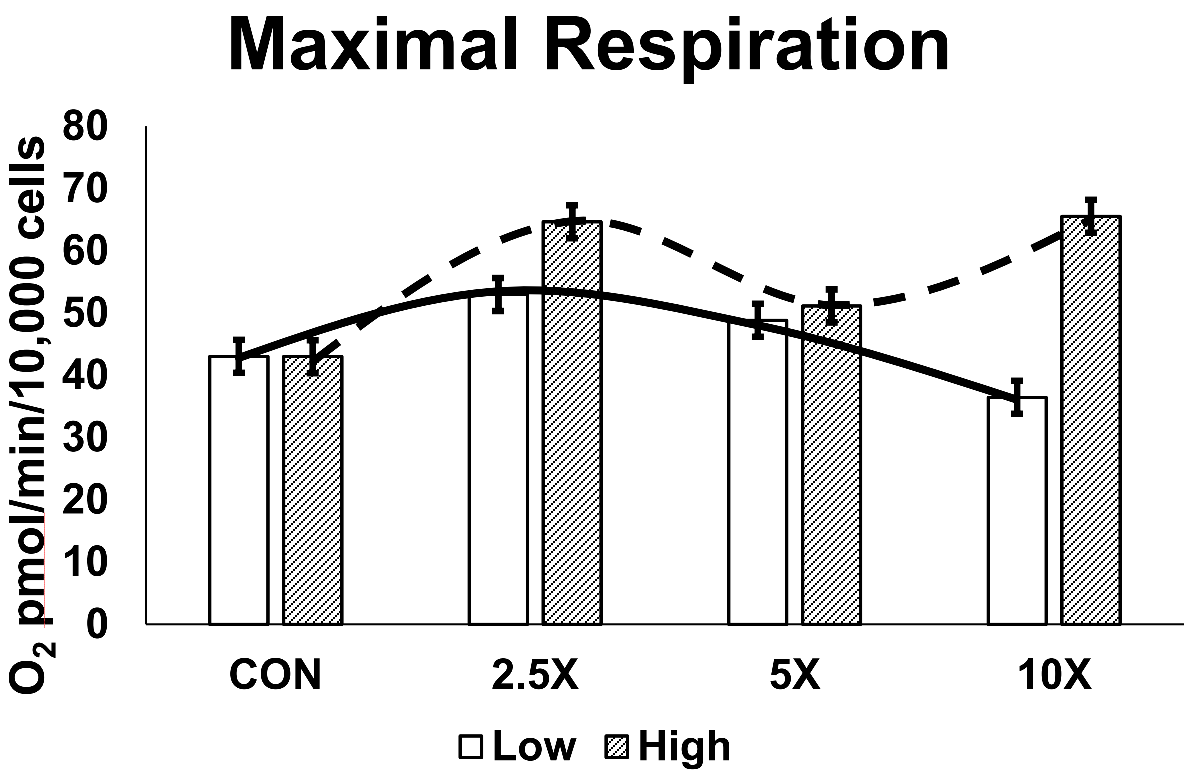
Maximal Respiration
O2 pmol/min/10,000 cells
80
70
60
50
40
30
20
10
0
CON
2.5X
5X
10X
Low
High

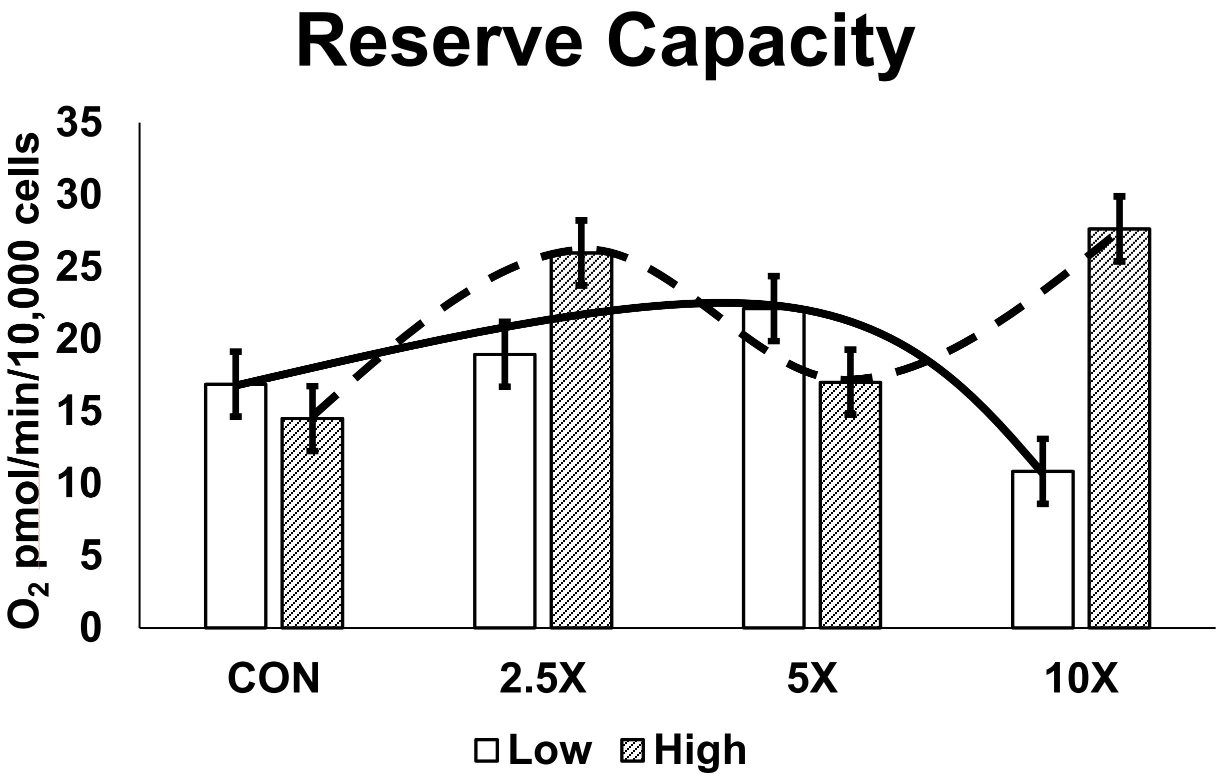
Reserve Capacity
O2 pmol/min/10,000 cells
35
30
25
20
15
10
5
0
CON
2.5X
5X
10X
Low
High

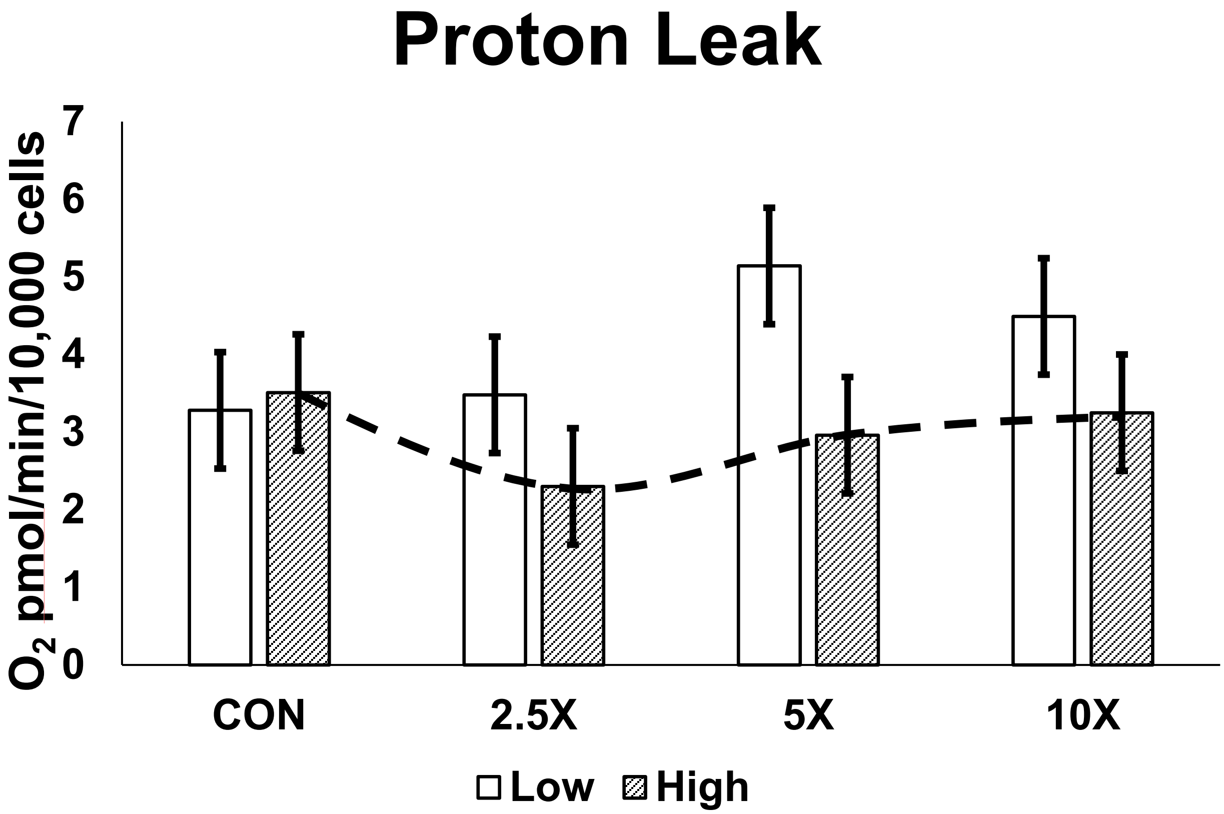
Proton Leak
7
6
5
4
3
2
1
0
O2 pmol/min/10,000 cells
CON
2.5X
5X
10X
Low
High

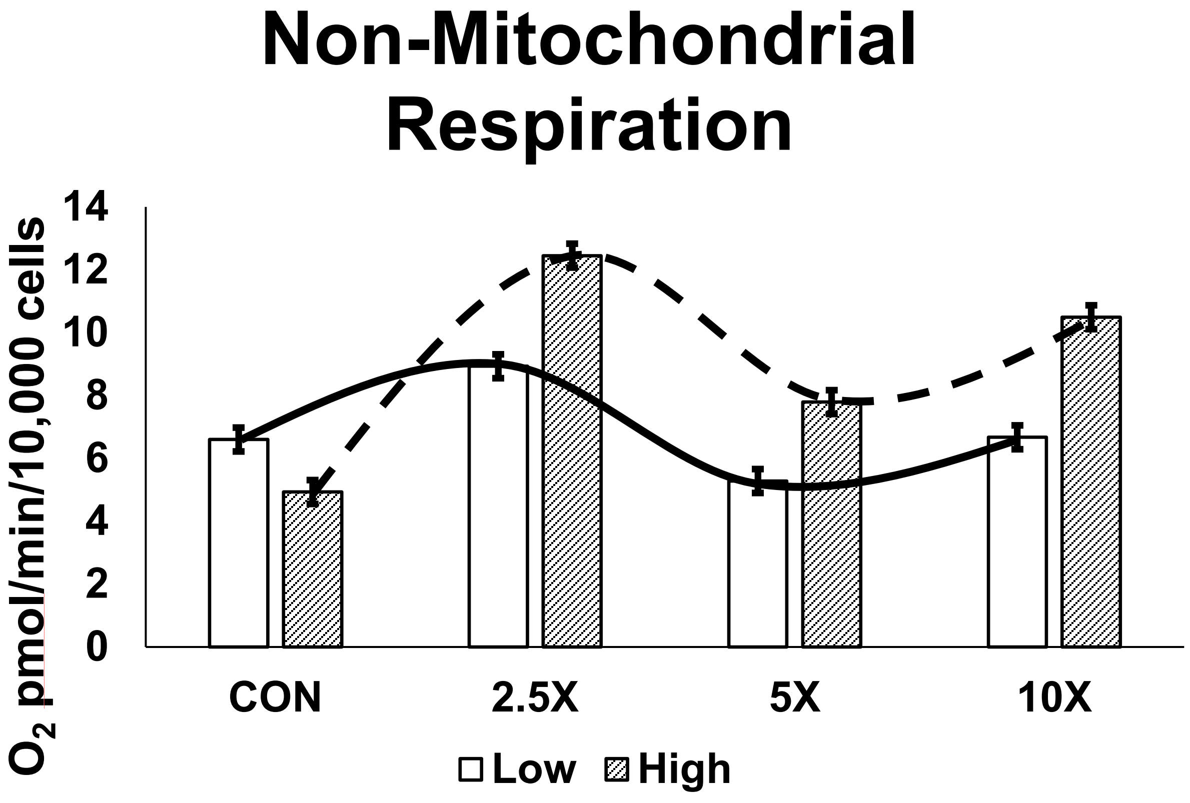
Non-Mitochondrial Respiration
14
12
10
8
6
4
2
0
O2 pmol/min/10,000 cells
CON
2.5X
5X
10X
Low
High

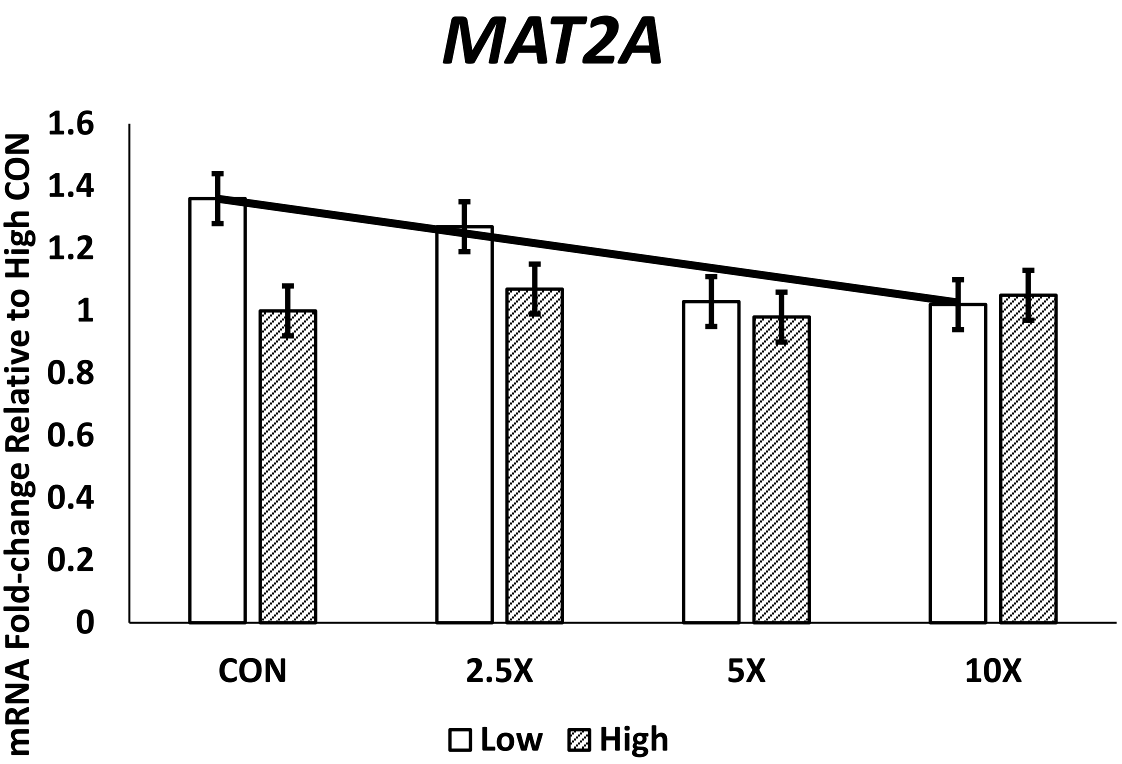
MAT2A
1.6
1.4
1.2
1
0.8
0.6
0.4
0.2
0
mRNA Fold-change Relative to High CON
CON
2.5X
5X
10X
Low
High

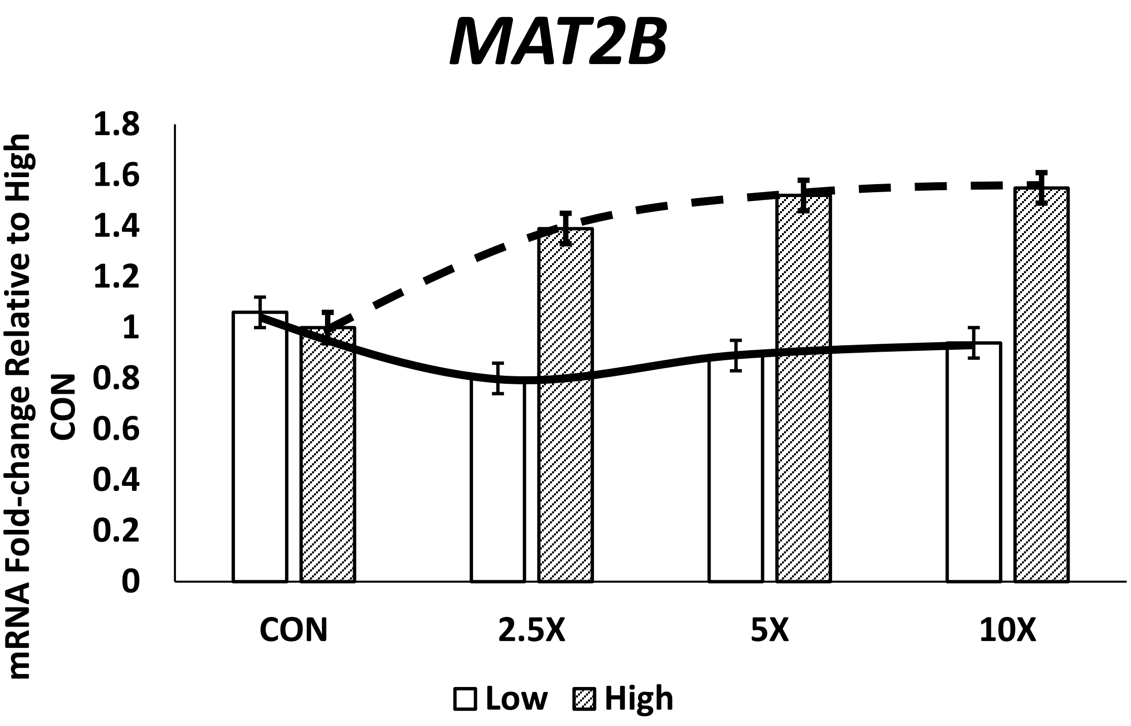
MAT2B
1.8
1.6
1.4
1.2
1
0.8
0.6
0.4
0.2
0
mRNA Fold-change Relative to High CON
CON
2.5X
5X
10X
Low
High

DNMT1

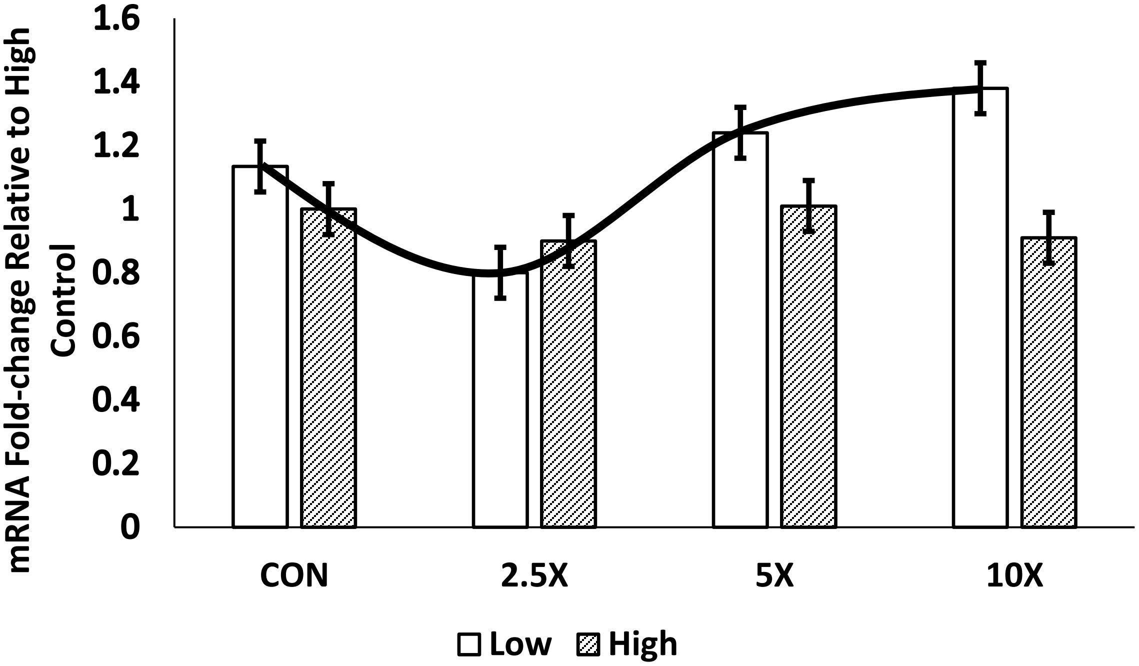

DNMT3B

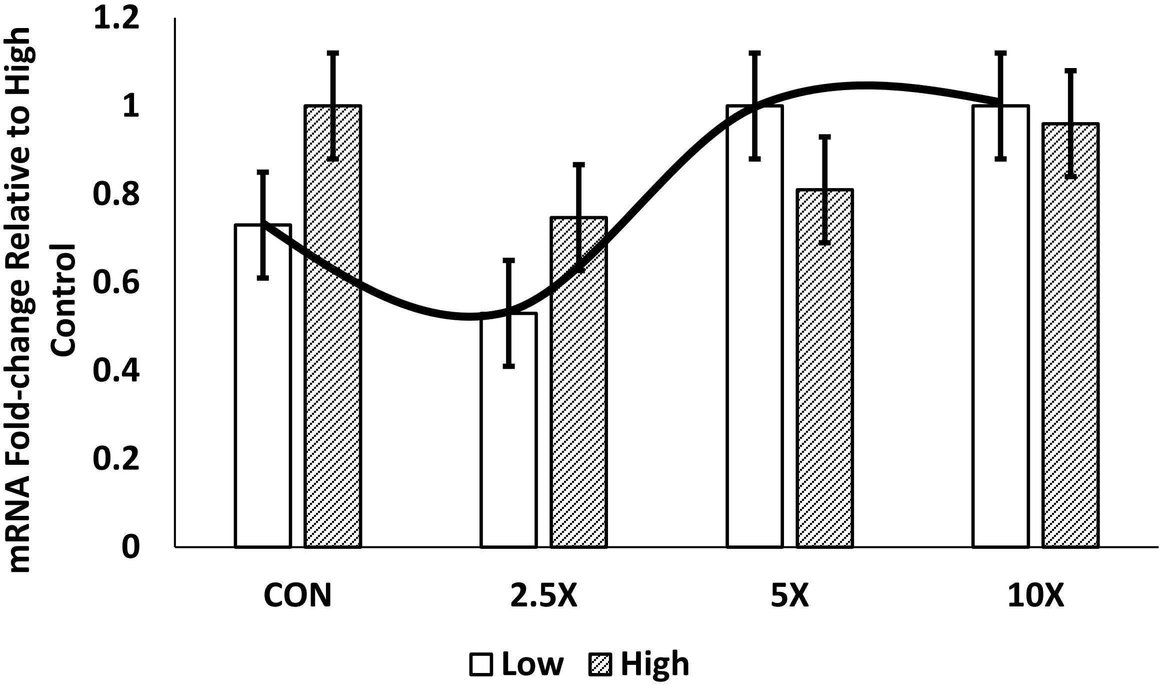

Supplement: Supplementary file 4 [file Image1.PDF]
